# Supplementary figures and images for: Impact of maximal overexpression of a non-toxic protein on yeast cell physiology
Source: eLife. 2025 Sep 17;13:RP99572. doi: 10.7554/eLife.99572 (PMC12443478; doi:10.7554/eLife.99572)

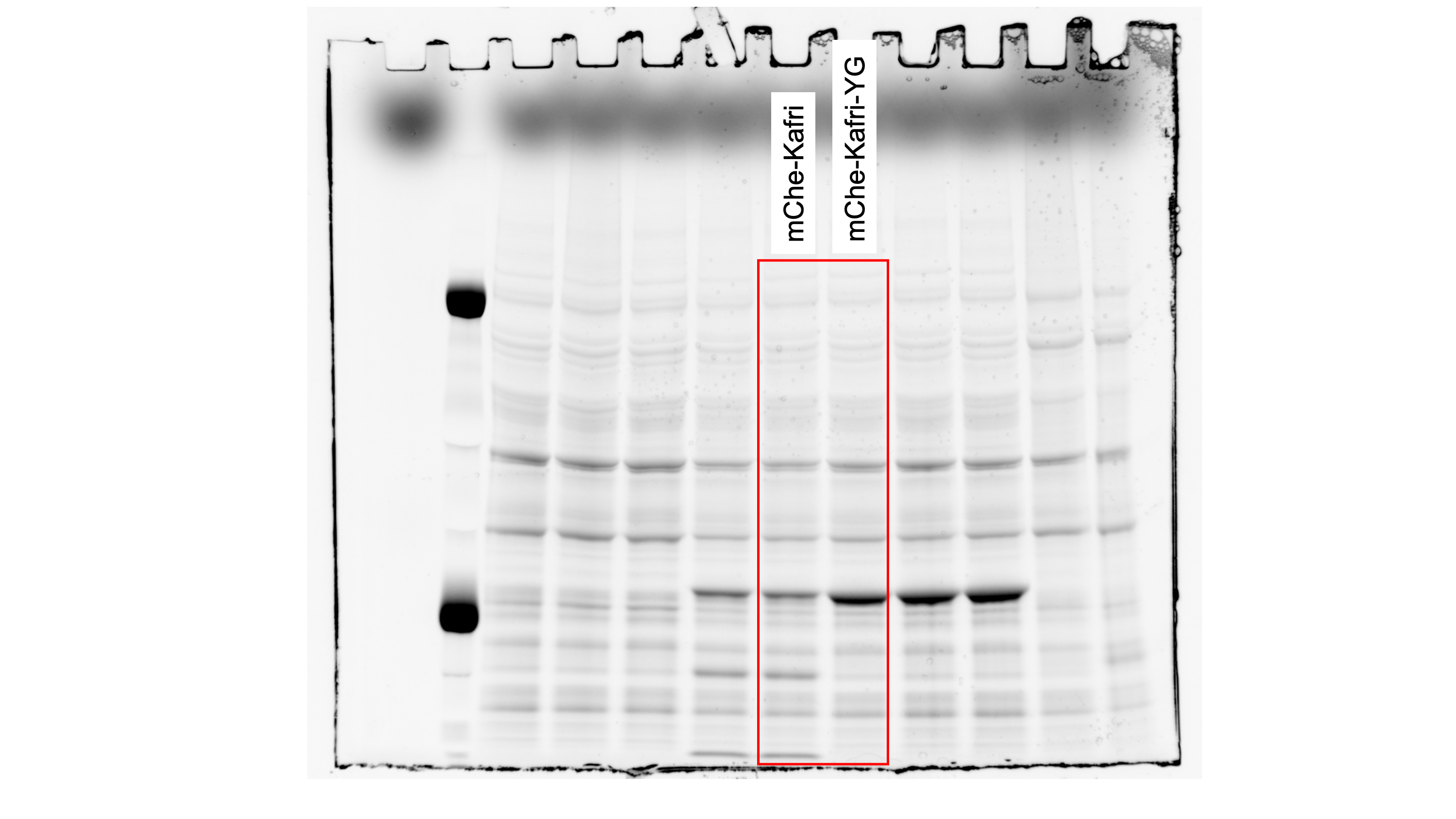

Supplement: Figure 2—source data 1. [file elife-99572-fig2-data1.zip › Figure 2ΓÇösource data 1/Figure 2ΓÇöfigure supplement 5C_Labelled.tiff]

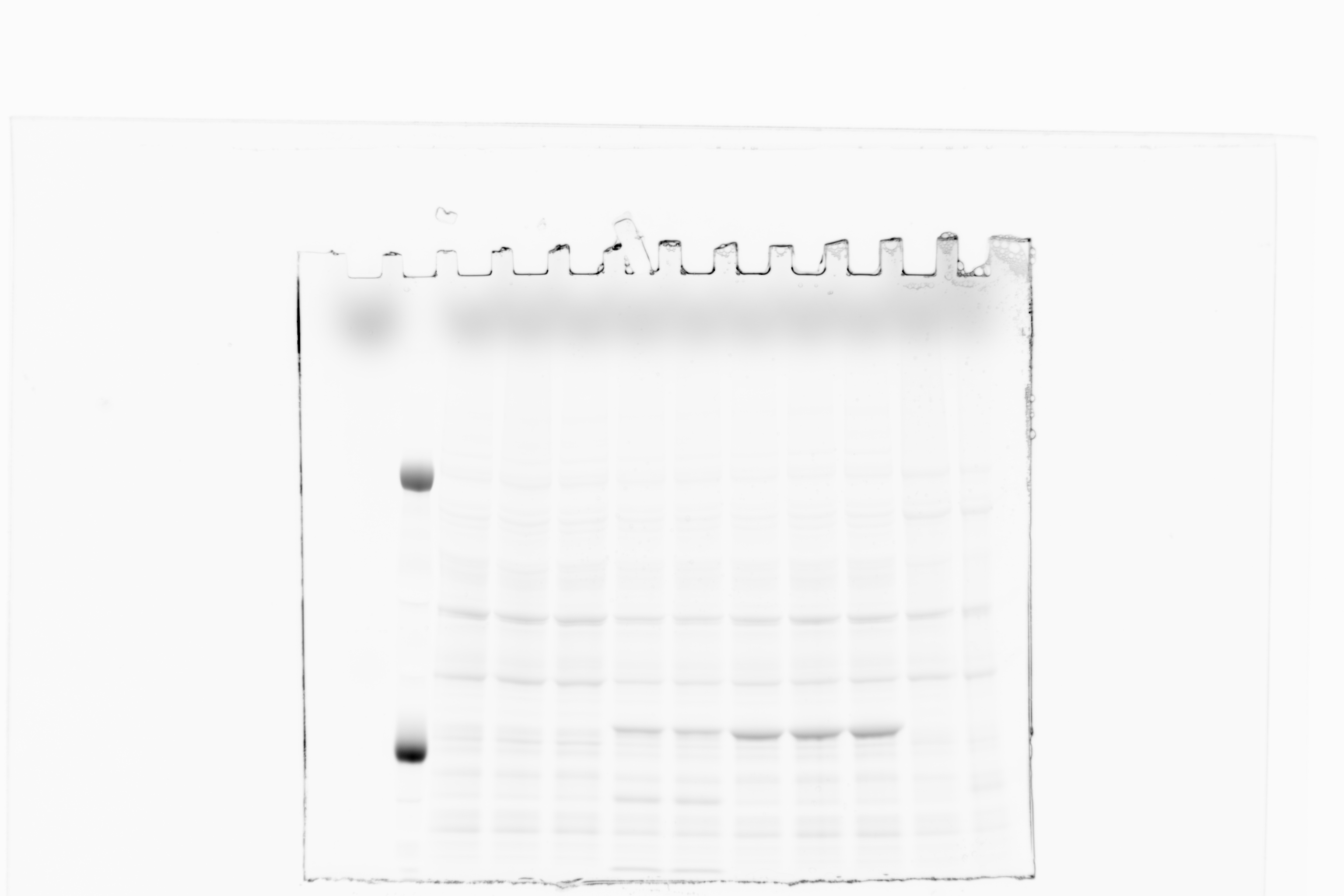

Supplement: Figure 2—source data 1. [file elife-99572-fig2-data1.zip › Figure 2ΓÇösource data 1/Figure 2ΓÇöfigure supplement 5C_Original.gel]

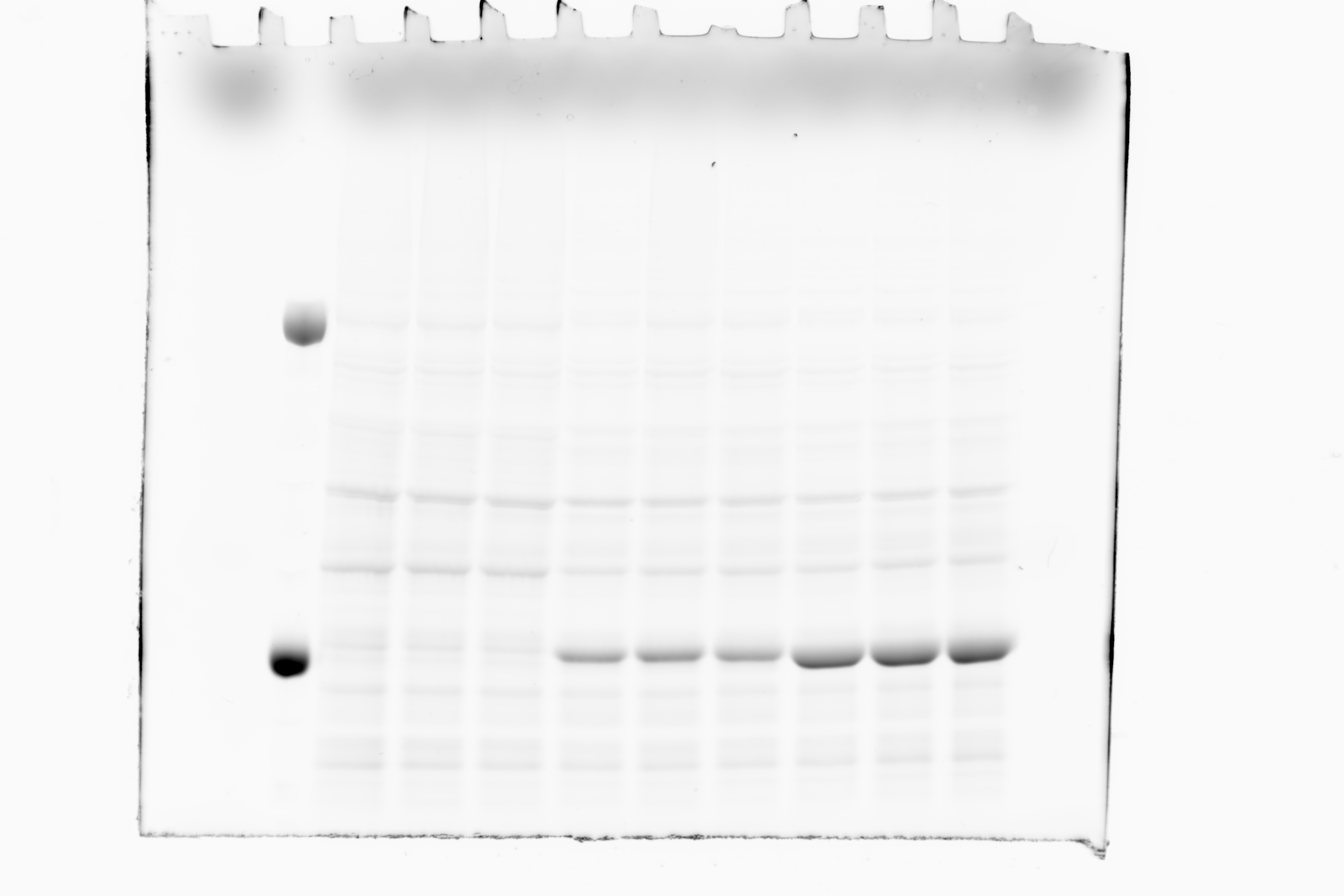

Supplement: Figure 2—source data 1. [file elife-99572-fig2-data1.zip › Figure 2ΓÇösource data 1/Figure 2ΓÇöfigure supplement 7G_Original.gel]

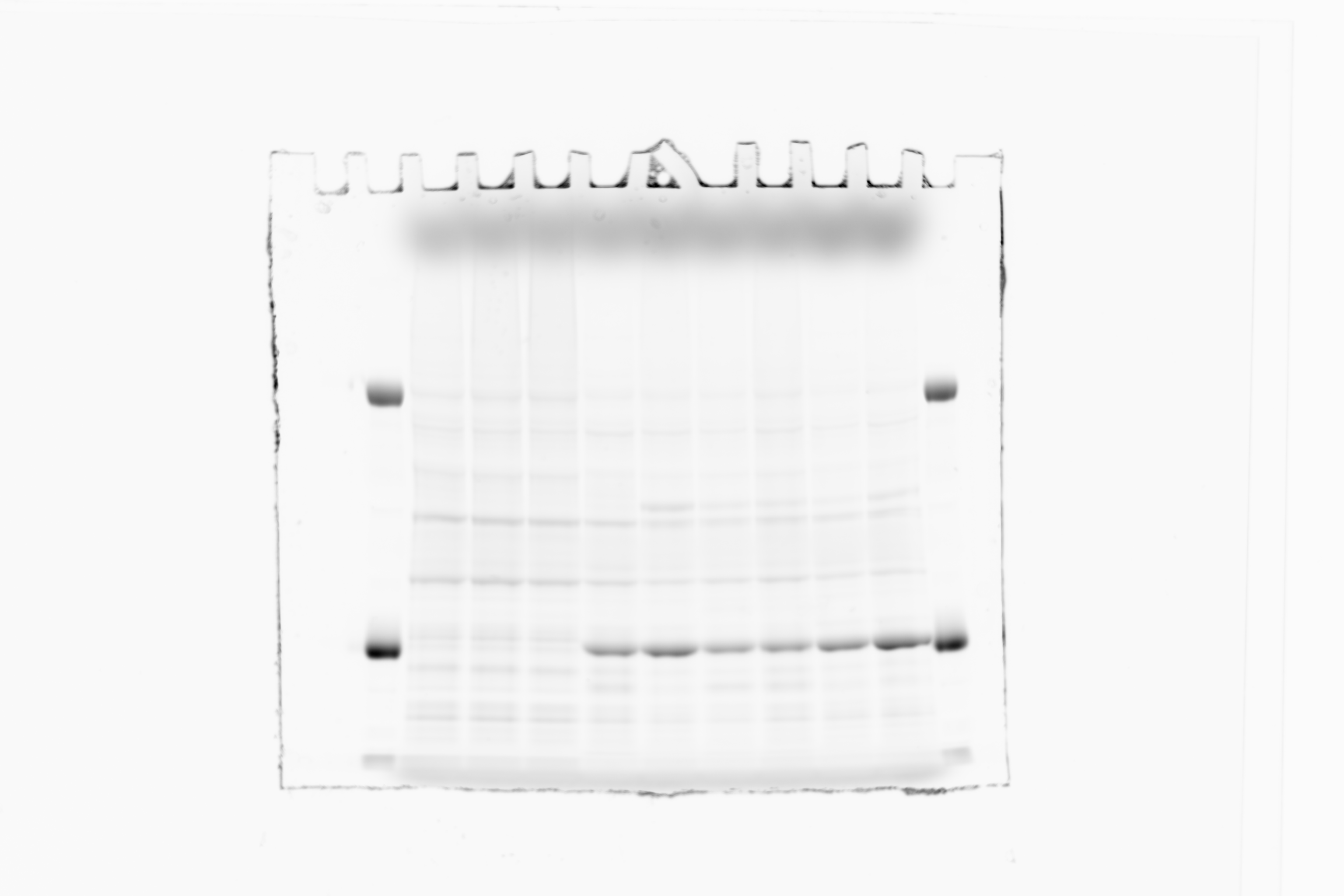

Supplement: Figure 2—source data 1. [file elife-99572-fig2-data1.zip › Figure 2ΓÇösource data 1/FIgure 2D_1_Original.gel]

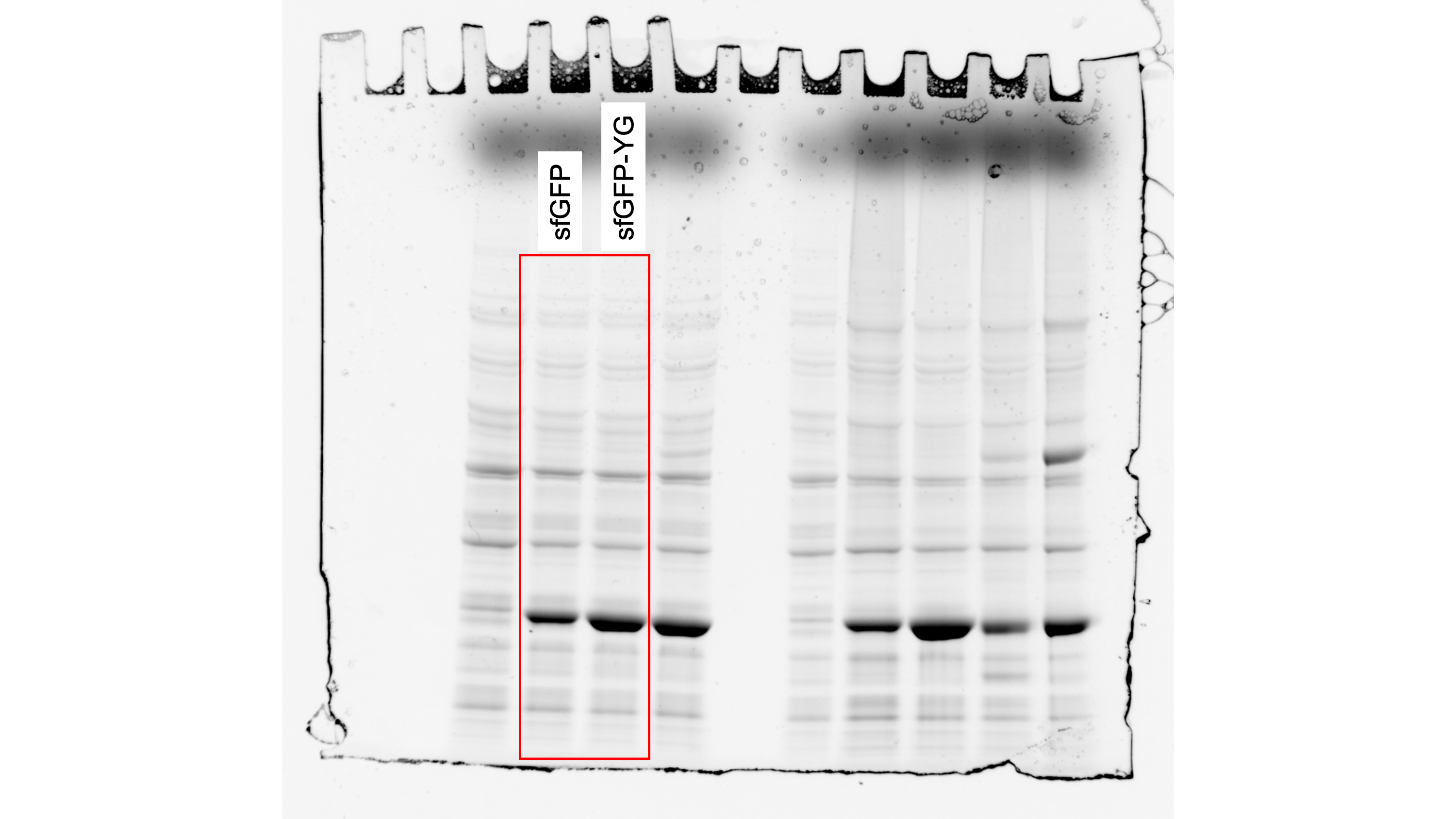

Supplement: Figure 2—source data 1. [file elife-99572-fig2-data1.zip › Figure 2ΓÇösource data 1/FIgure 2D_2_Labelled.tiff]

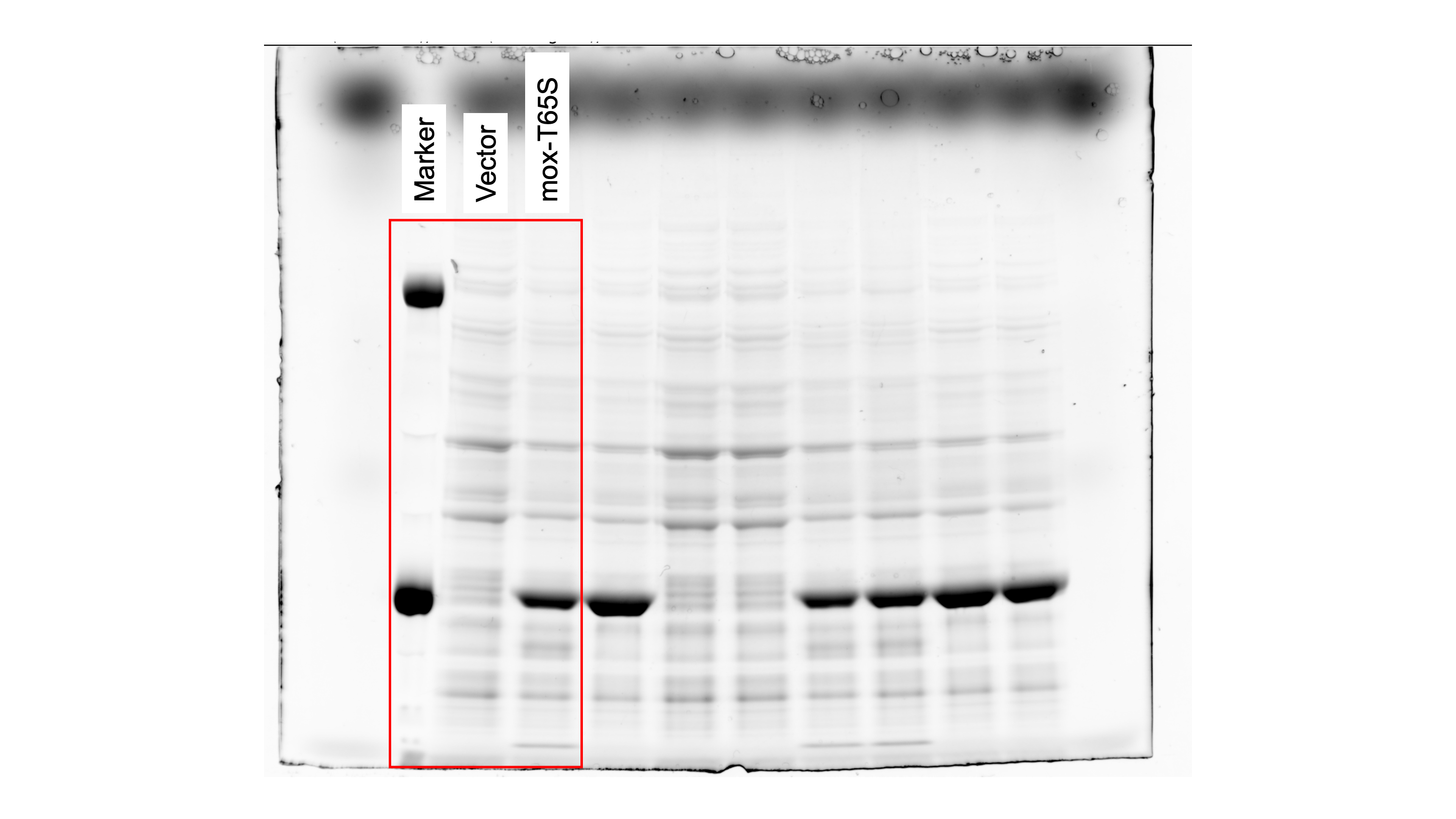

Supplement: Figure 2—source data 1. [file elife-99572-fig2-data1.zip › Figure 2ΓÇösource data 1/Figure 2ΓÇöfigure supplement 10H_Labelled.tiff]

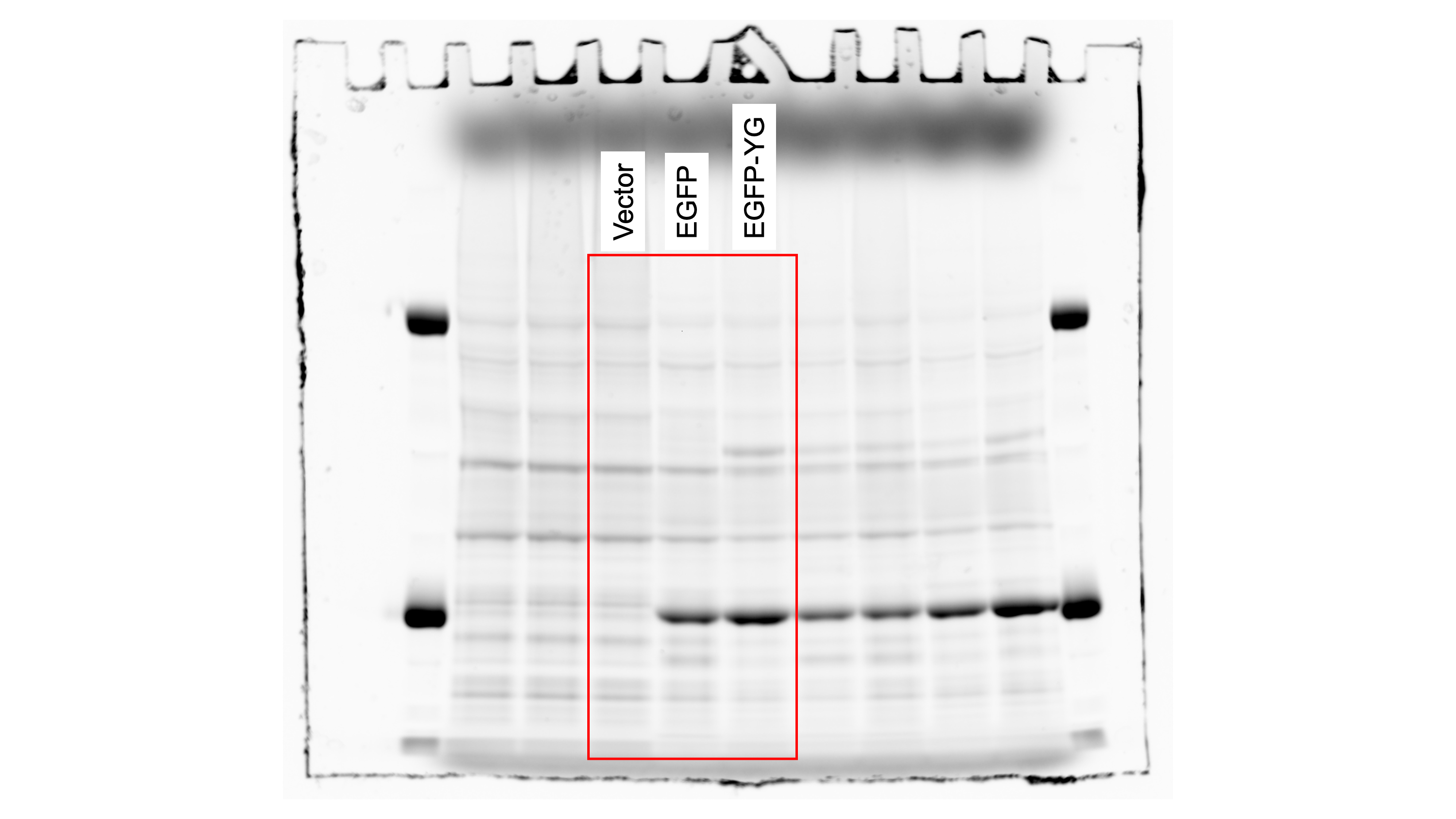

Supplement: Figure 2—source data 1. [file elife-99572-fig2-data1.zip › Figure 2ΓÇösource data 1/FIgure 2D_1_Labelled.tiff]

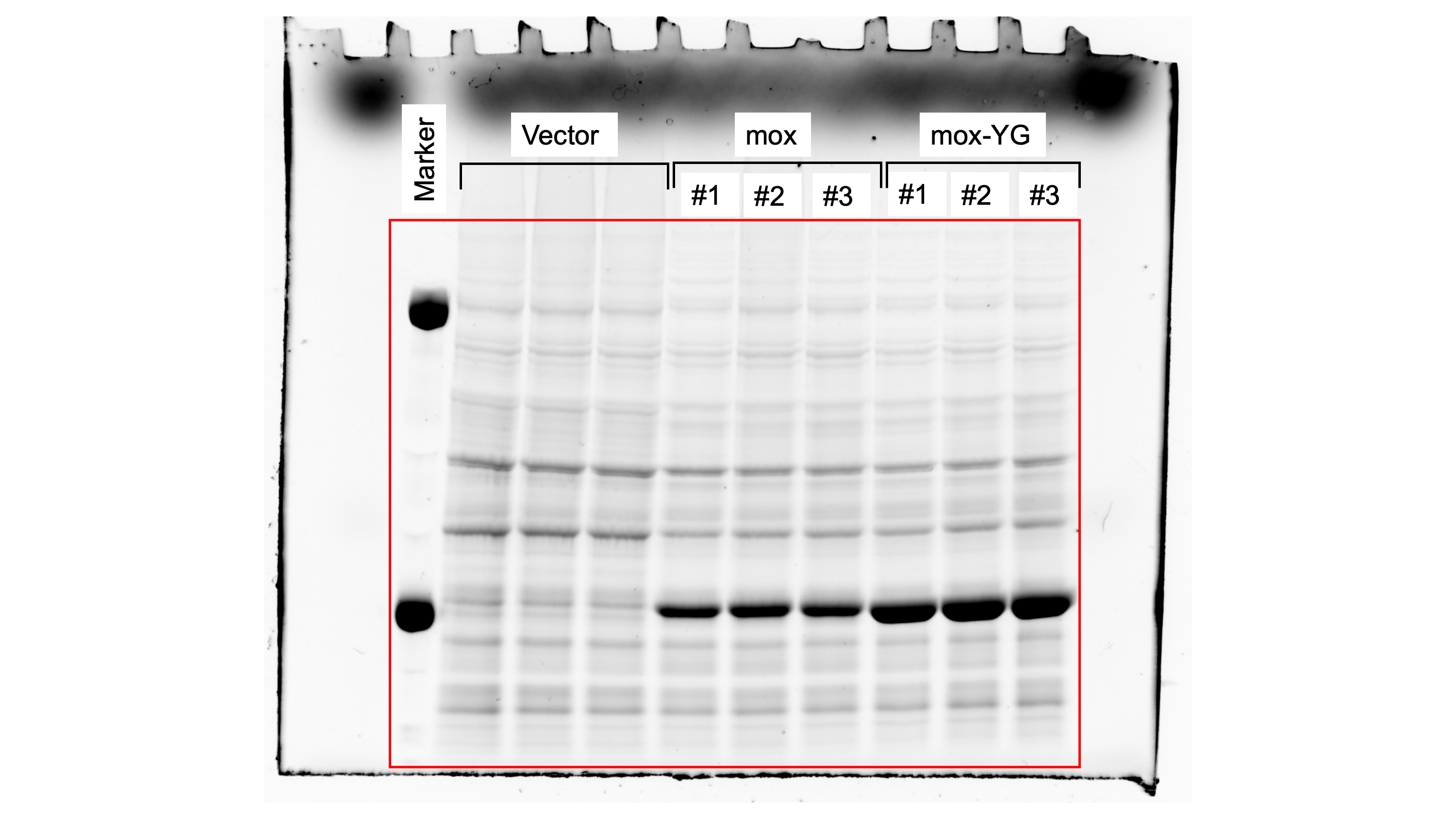

Supplement: Figure 2—source data 1. [file elife-99572-fig2-data1.zip › Figure 2ΓÇösource data 1/Figure 2ΓÇöfigure supplement 7G_Labelled.tiff]

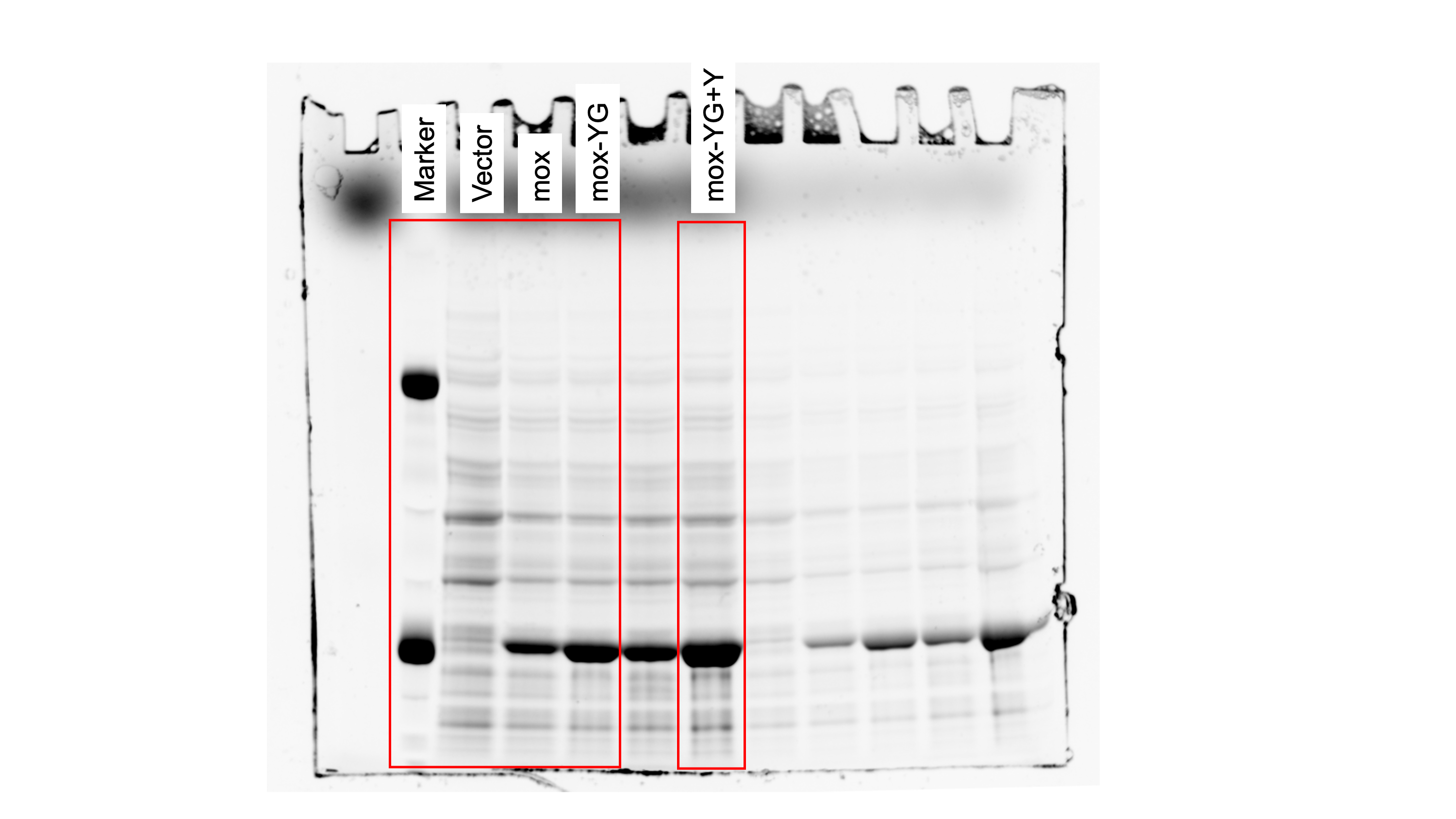

Supplement: Figure 2—source data 1. [file elife-99572-fig2-data1.zip › Figure 2ΓÇösource data 1/Figure 2ΓÇöfigure supplement 8B_Labelled.tiff]

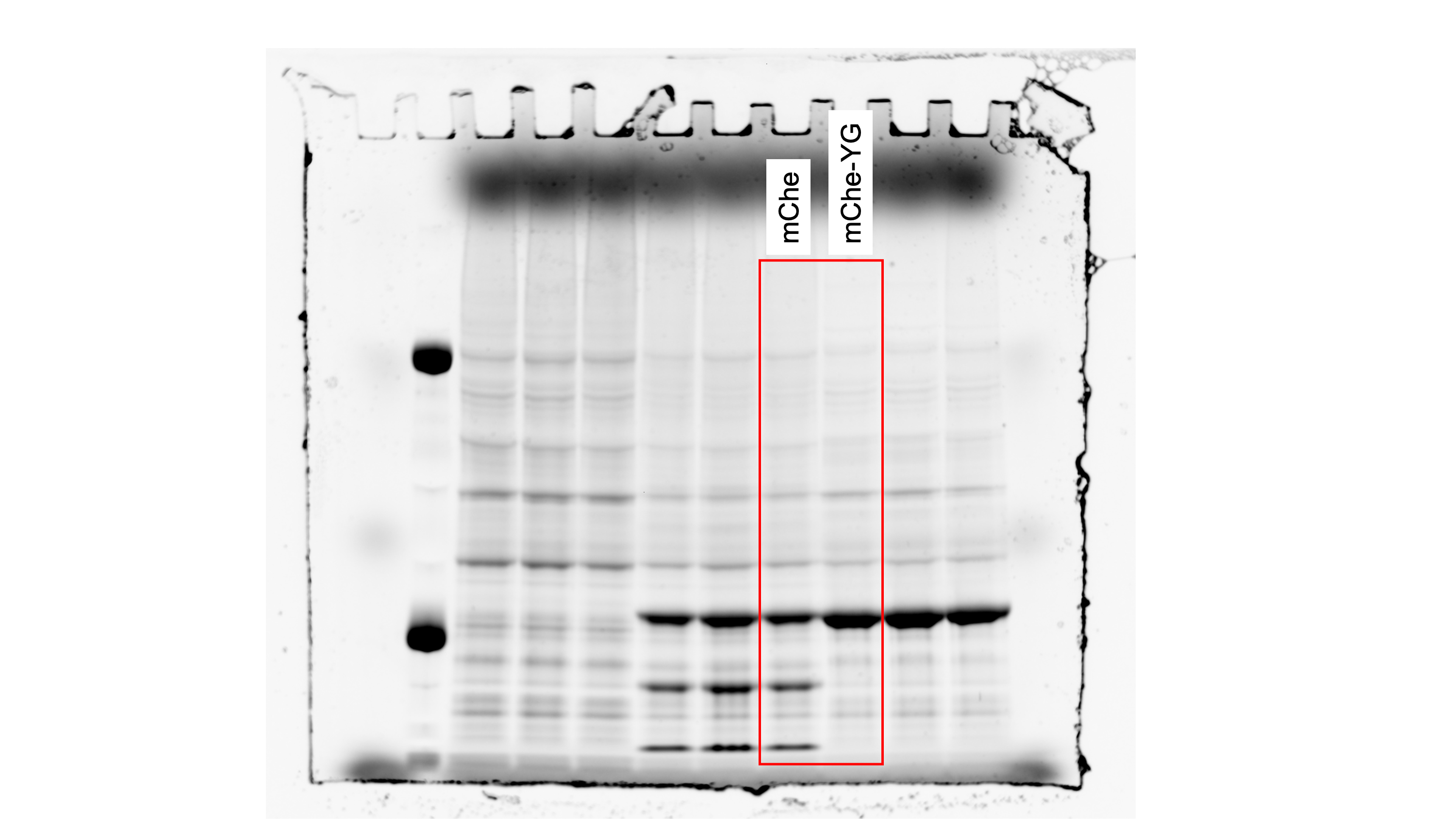

Supplement: Figure 2—source data 1. [file elife-99572-fig2-data1.zip › Figure 2ΓÇösource data 1/FIgure 2D_4_Labelled.tiff]

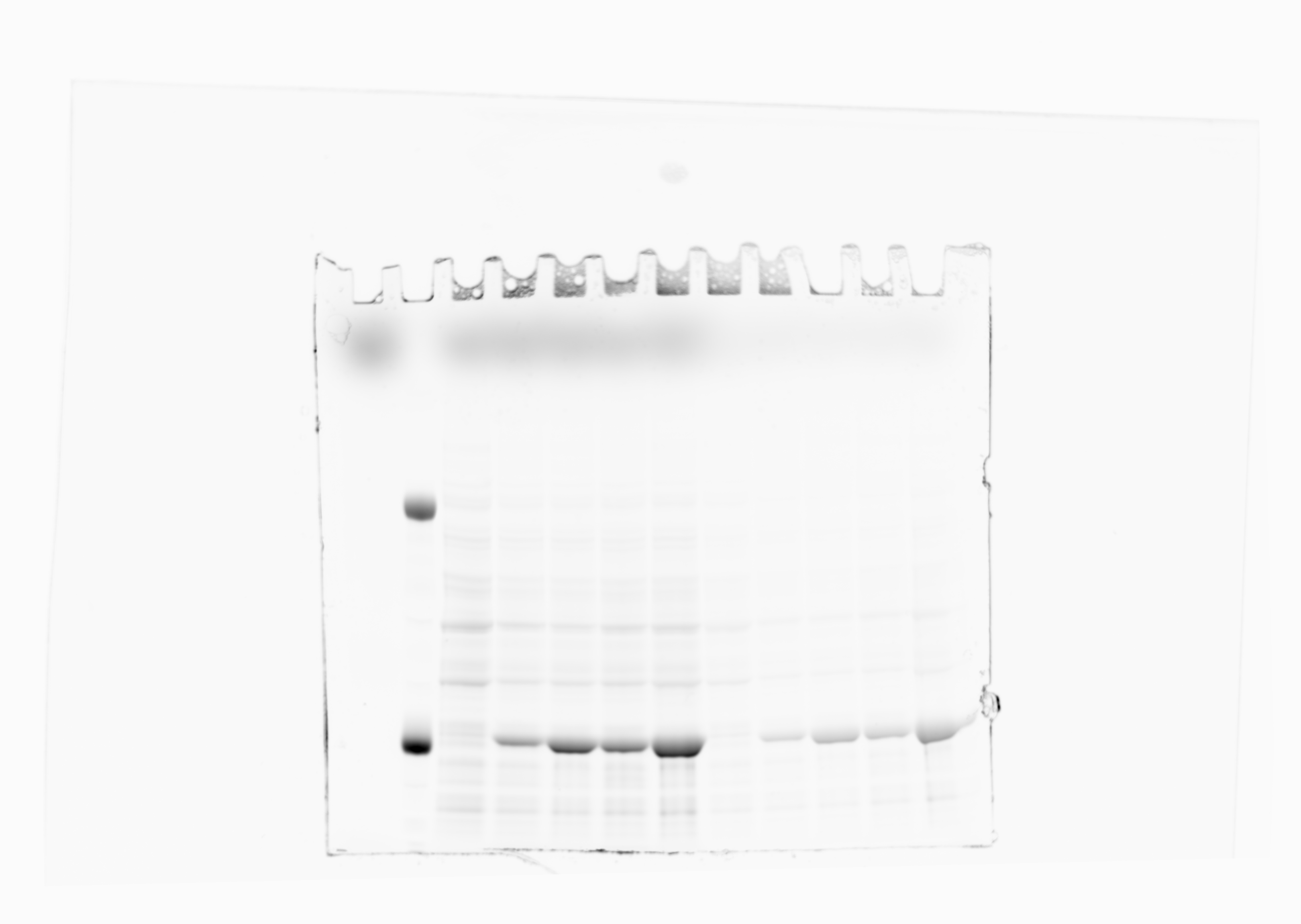

Supplement: Figure 2—source data 1. [file elife-99572-fig2-data1.zip › Figure 2ΓÇösource data 1/Figure 2ΓÇöfigure supplement 8B_10D_Original.gel]

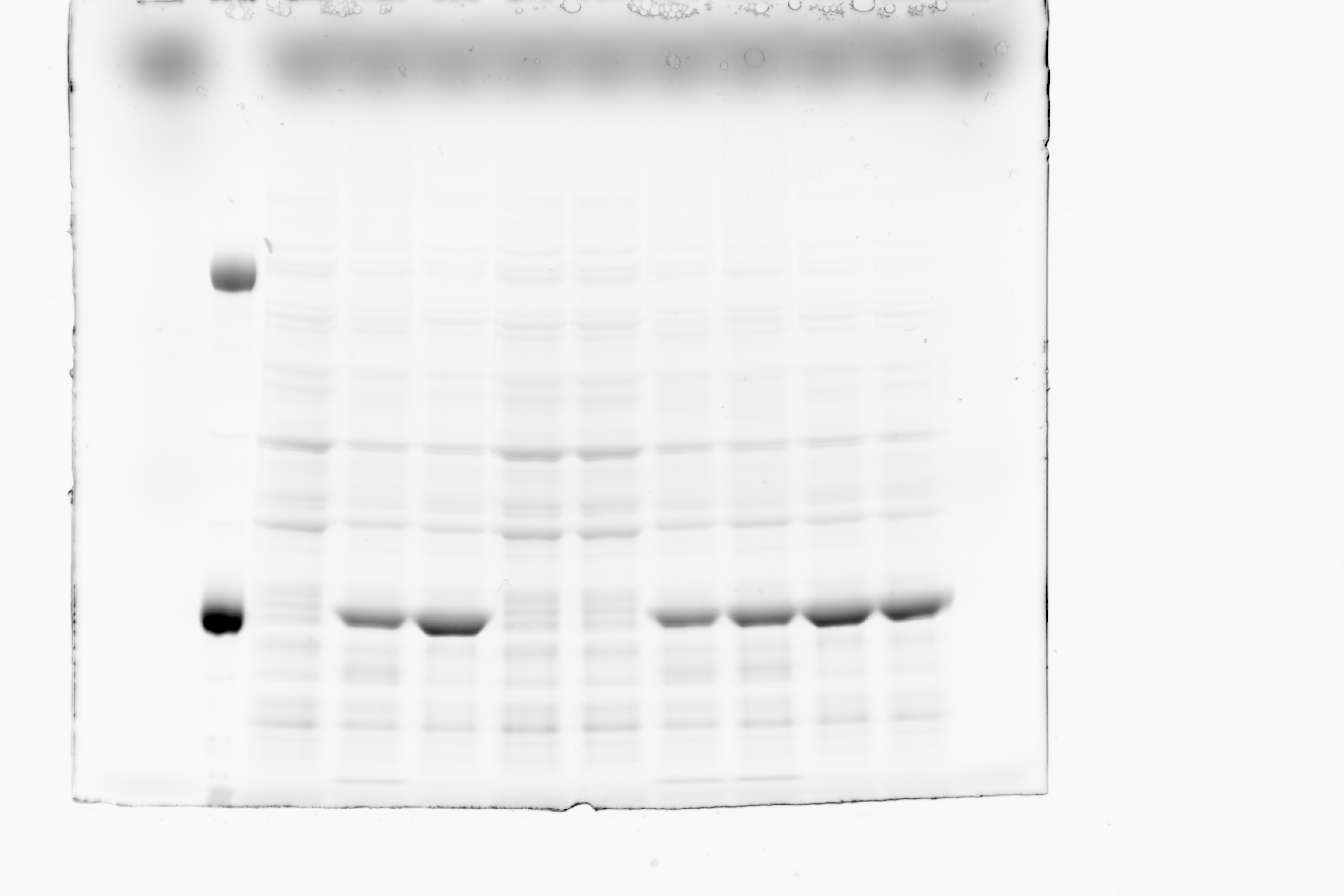

Supplement: Figure 2—source data 1. [file elife-99572-fig2-data1.zip › Figure 2ΓÇösource data 1/Figure 2ΓÇöfigure supplement 10H_Original.gel]

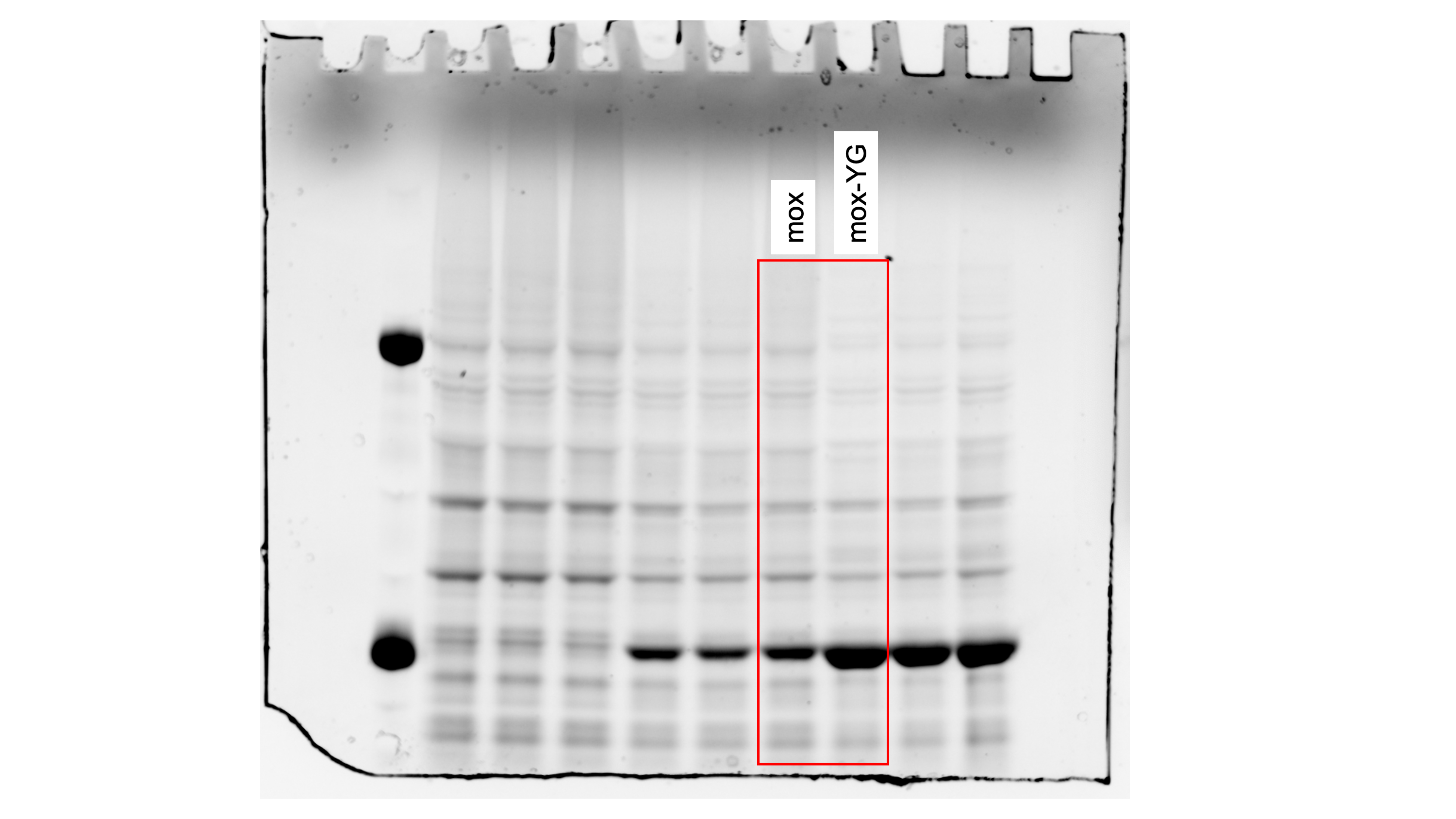

Supplement: Figure 2—source data 1. [file elife-99572-fig2-data1.zip › Figure 2ΓÇösource data 1/FIgure 2D_3_Labelled.tiff]

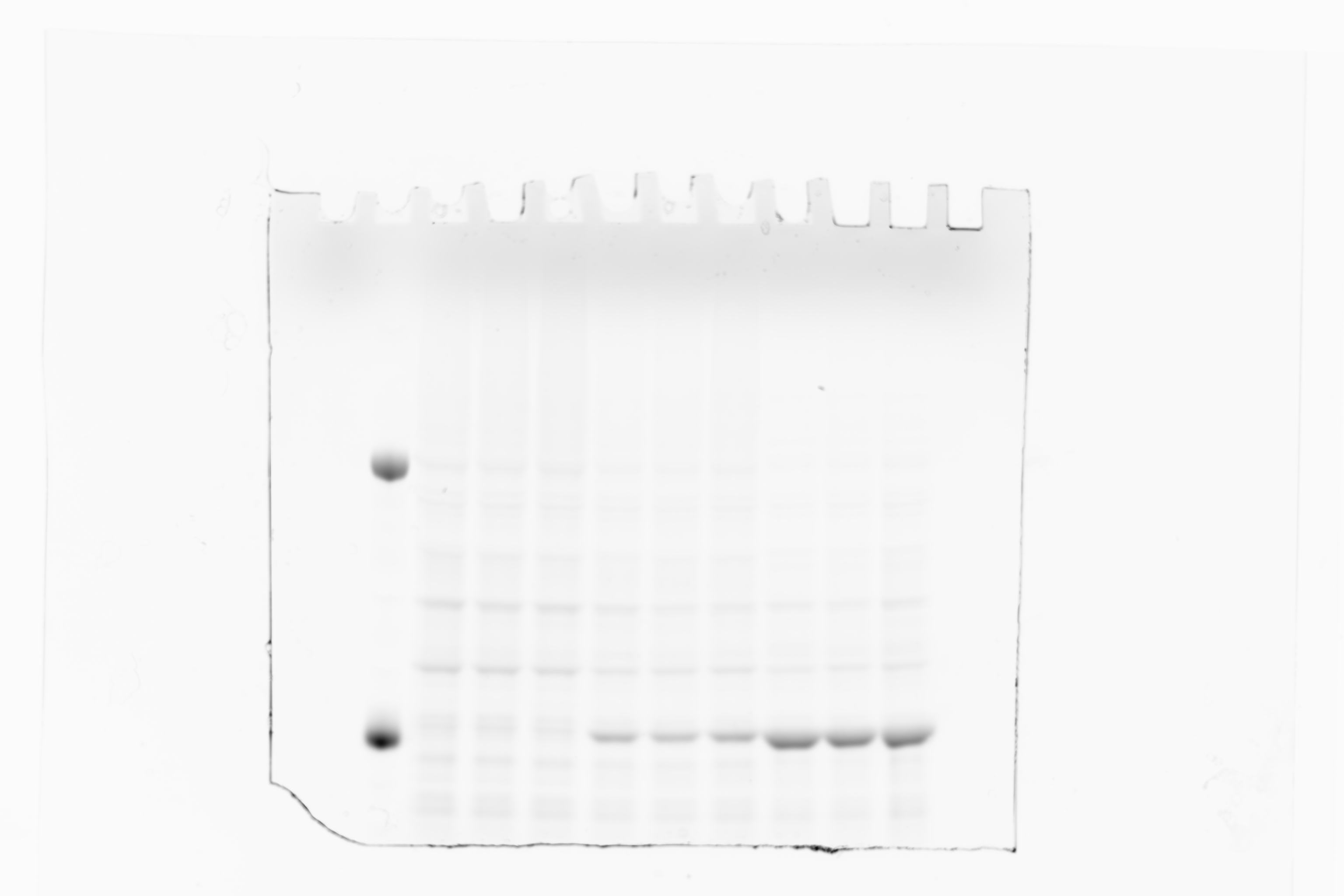

Supplement: Figure 2—source data 1. [file elife-99572-fig2-data1.zip › Figure 2ΓÇösource data 1/FIgure 2D_3_Original.gel]

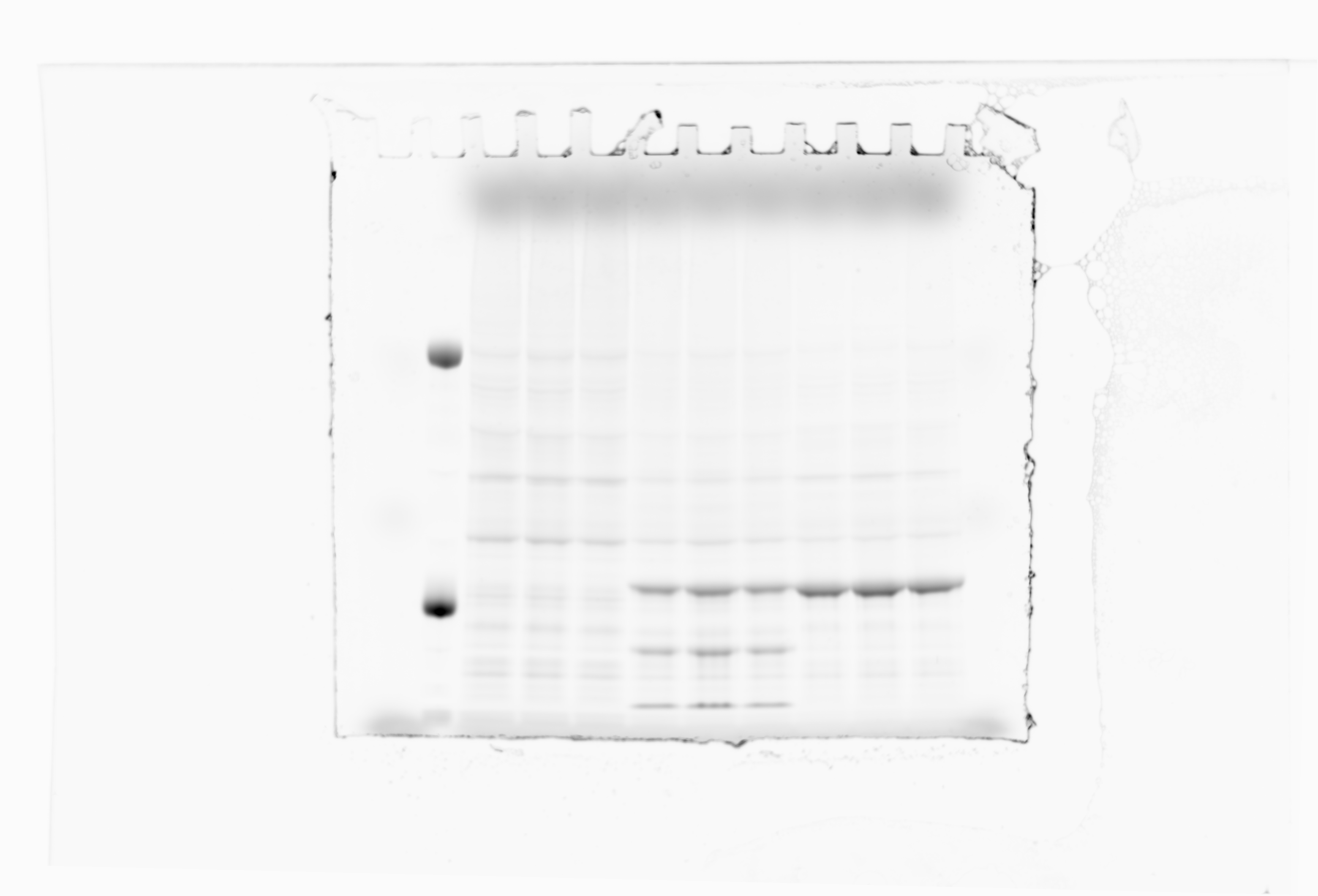

Supplement: Figure 2—source data 1. [file elife-99572-fig2-data1.zip › Figure 2ΓÇösource data 1/FIgure 2D_4_Original.gel]

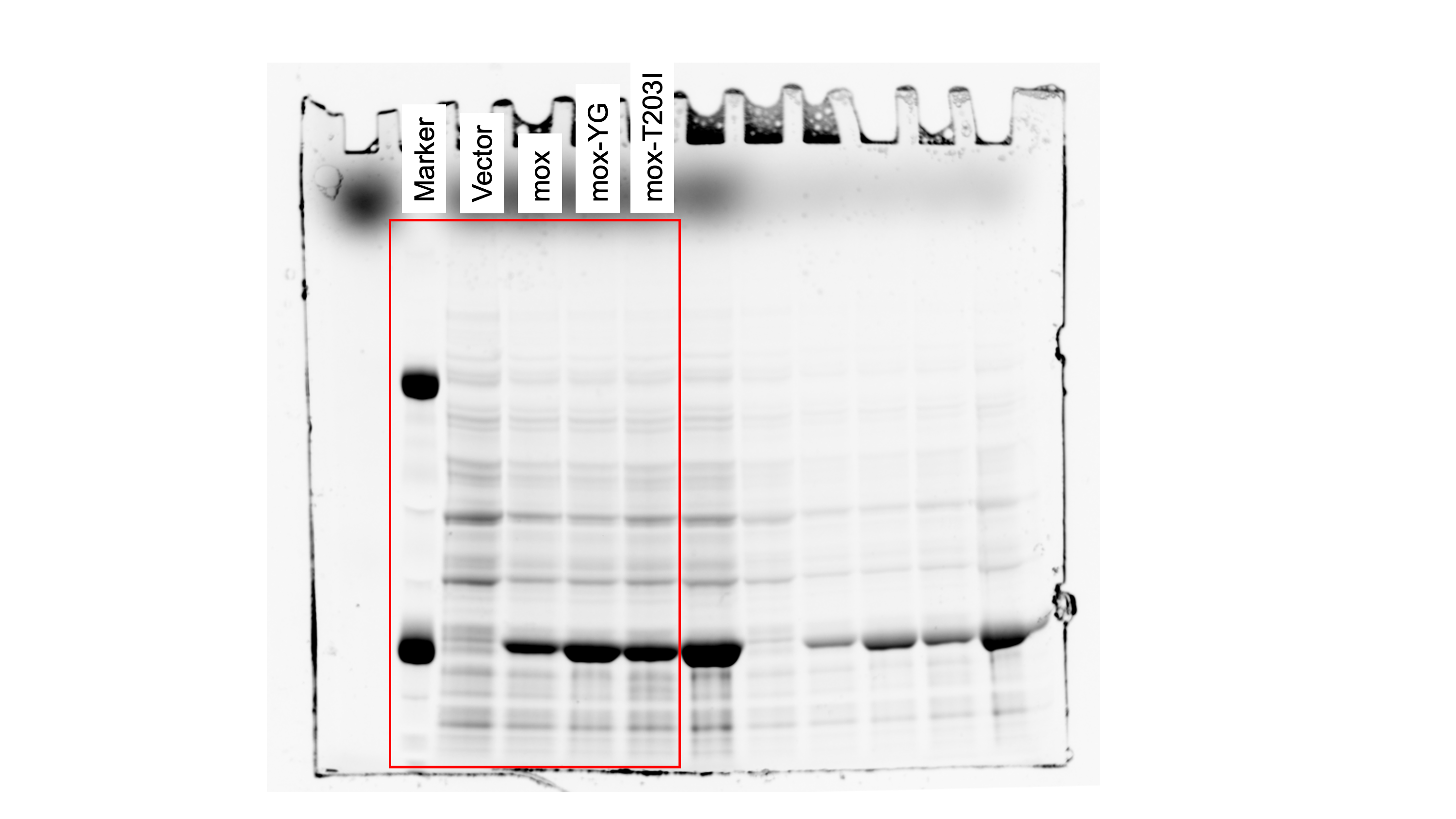

Supplement: Figure 2—source data 1. [file elife-99572-fig2-data1.zip › Figure 2ΓÇösource data 1/Figure 2ΓÇöfigure supplement 10D_Labelled.tiff]

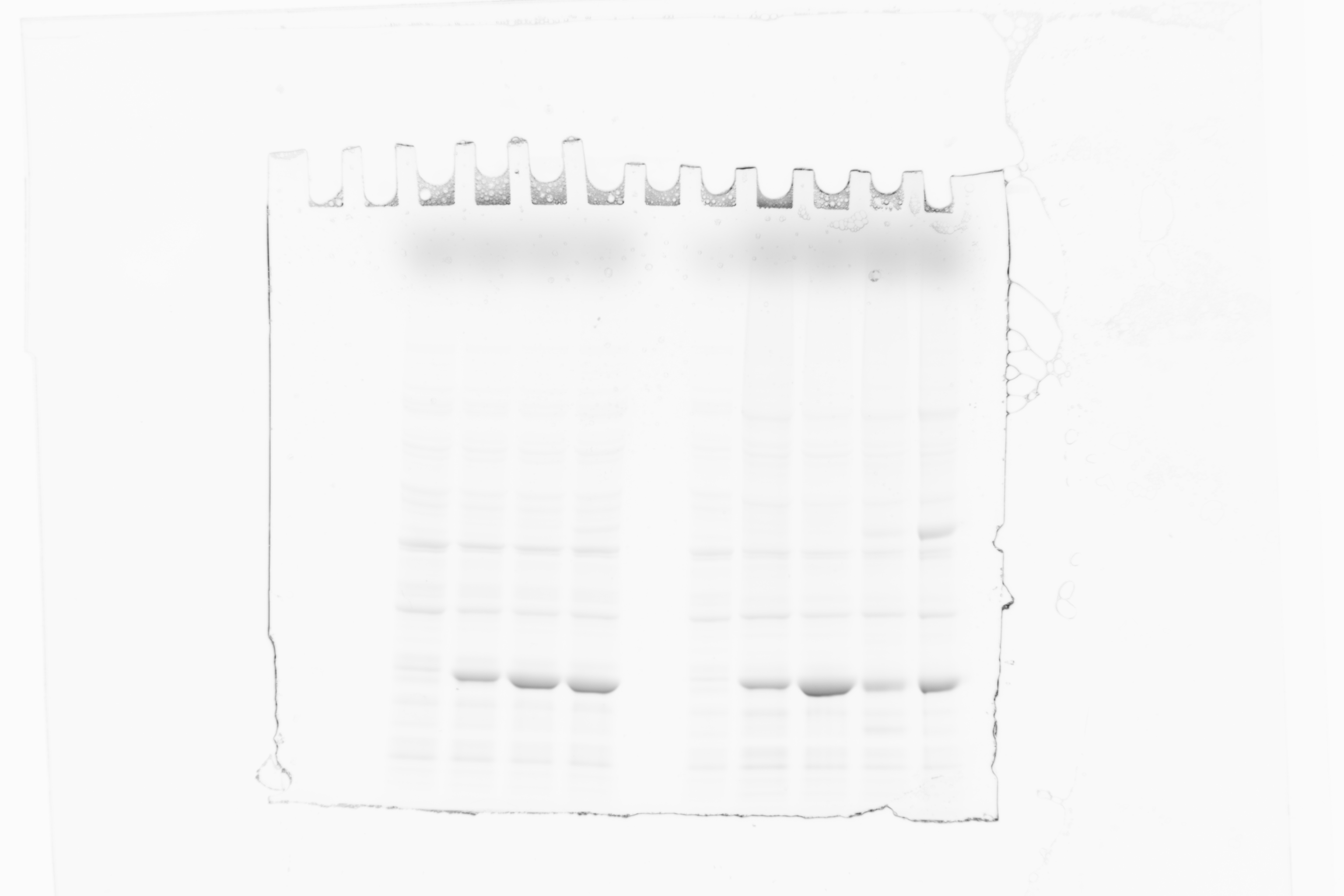

Supplement: Figure 2—source data 1. [file elife-99572-fig2-data1.zip › Figure 2ΓÇösource data 1/FIgure 2D_2_Original.gel]

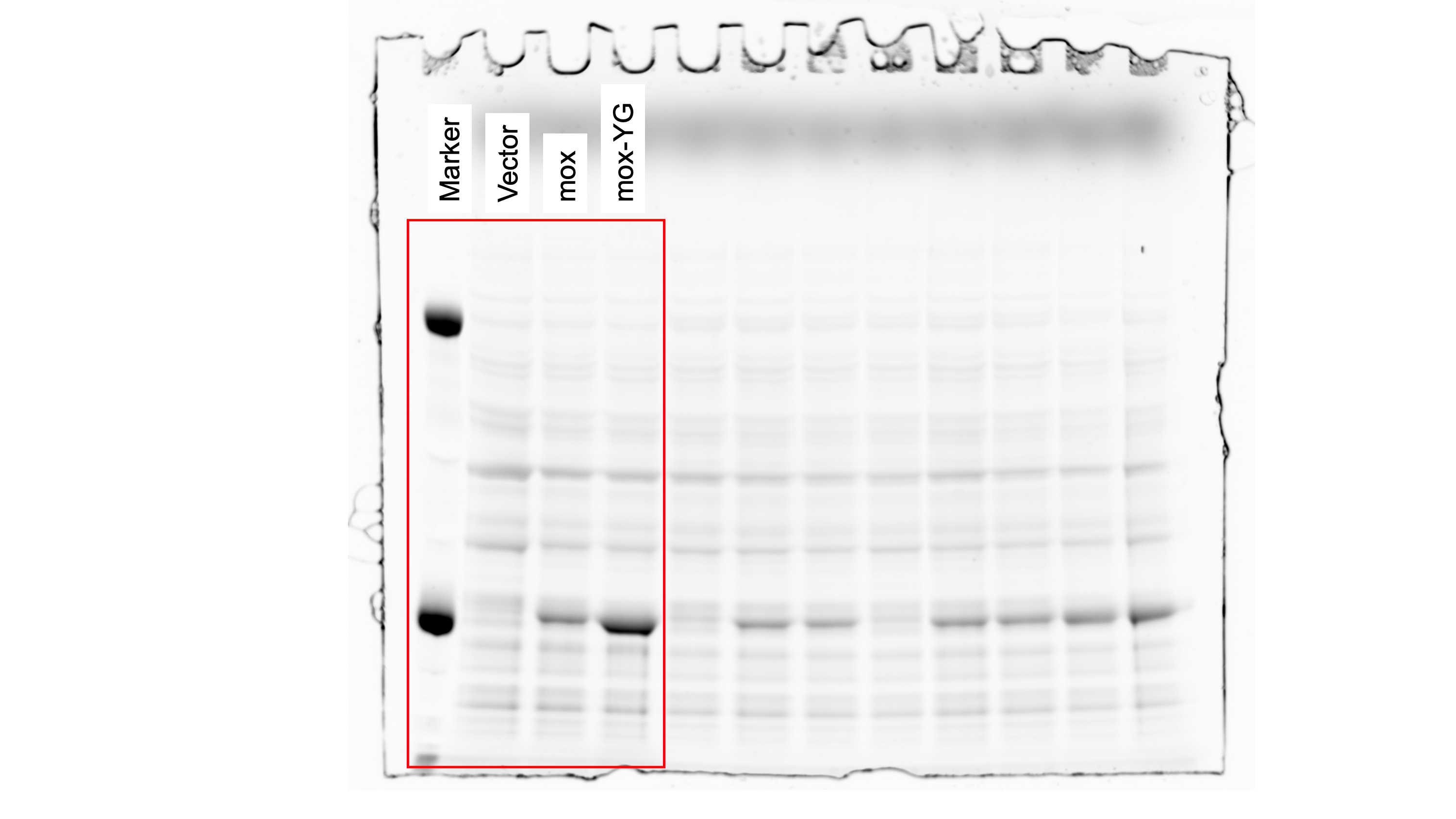

Supplement: Figure 4—figure supplement 2—source data 1. [file elife-99572-fig4-figsupp2-data1.zip › Figure 4ΓÇösource data 1/Figure 4ΓÇöfigure supplement 2C_Labelled.tiff]

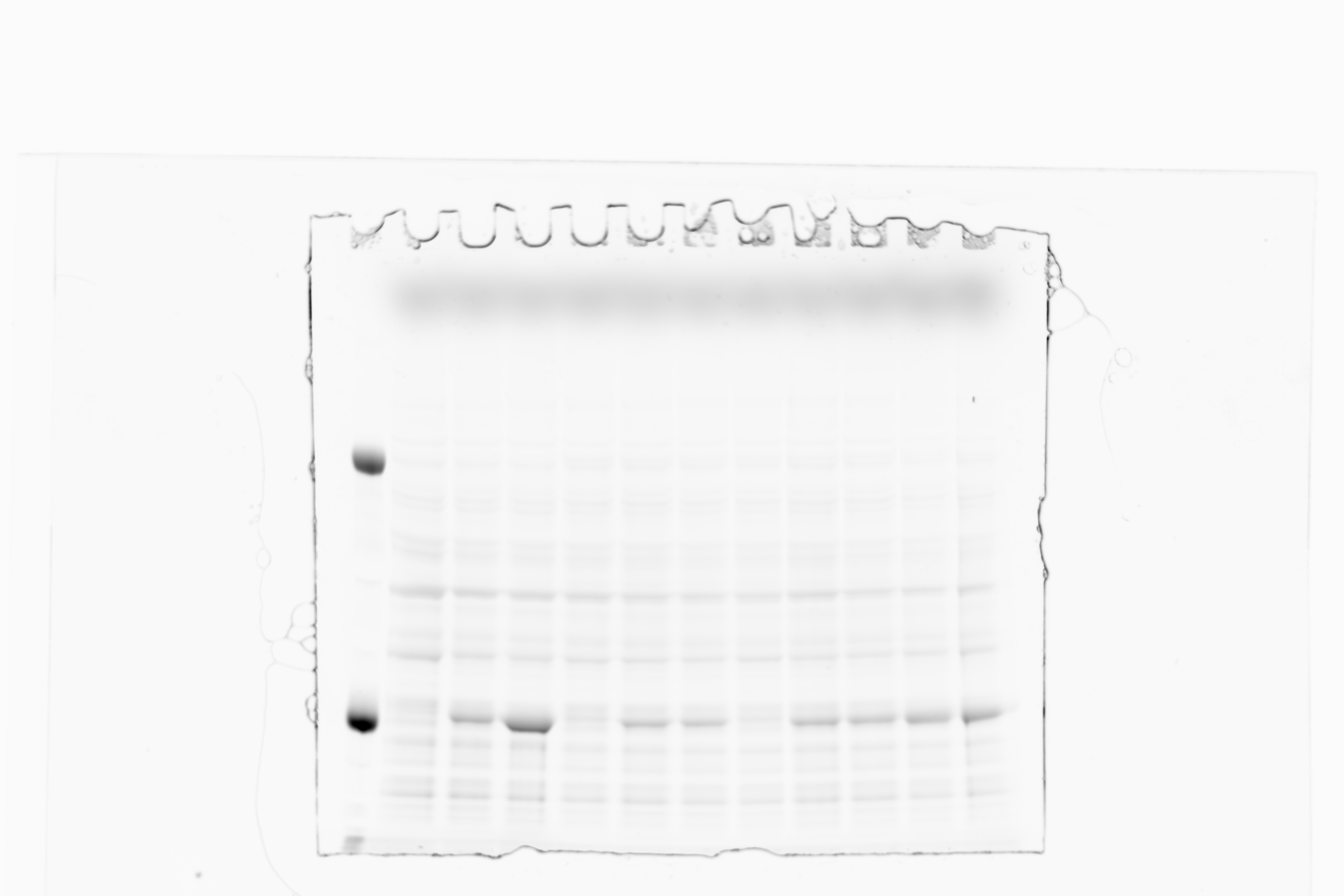

Supplement: Figure 4—figure supplement 2—source data 1. [file elife-99572-fig4-figsupp2-data1.zip › Figure 4ΓÇösource data 1/Figure 4ΓÇöfigure supplement 2C_Original.gel]

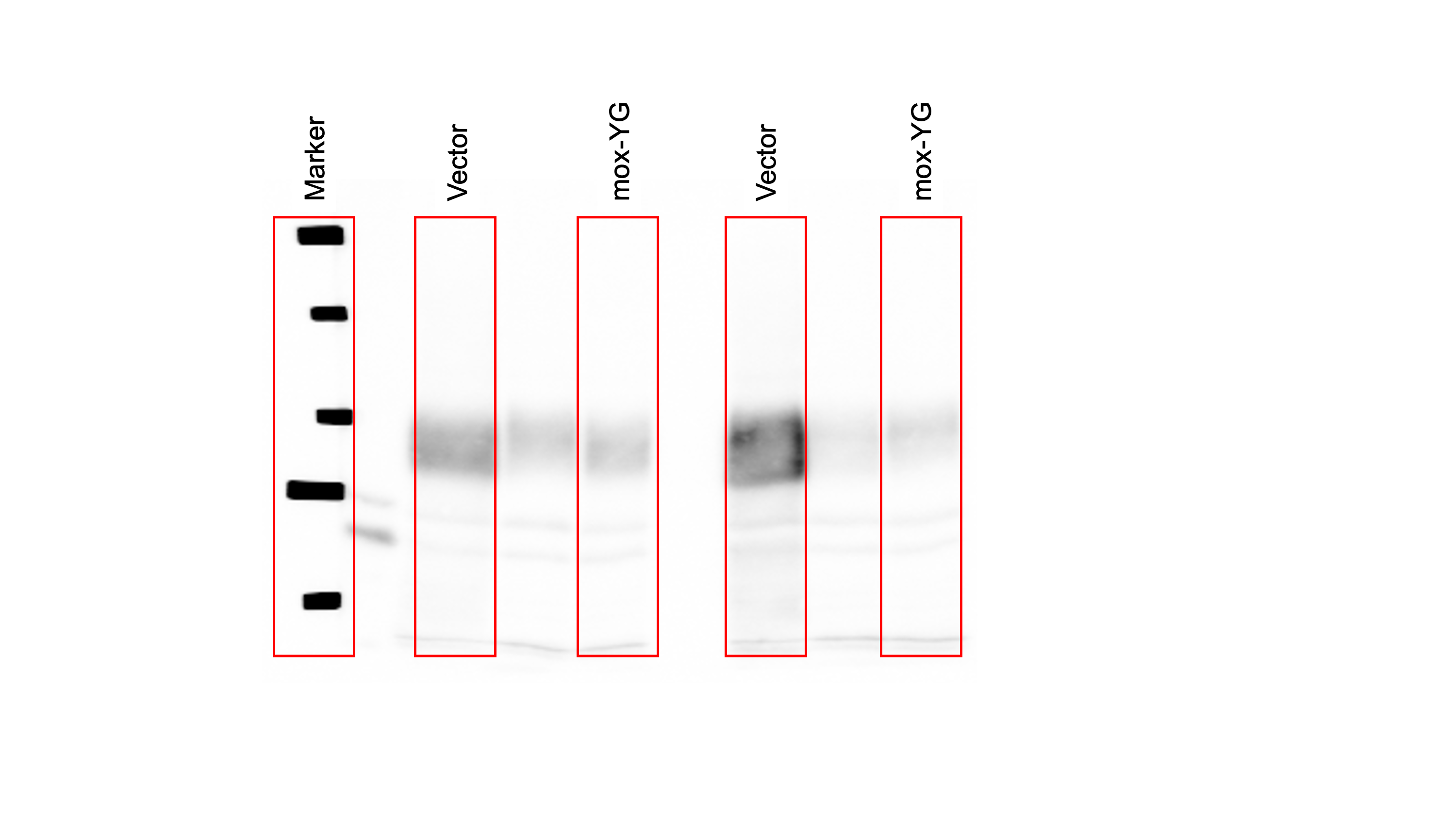

Supplement: Figure 4—figure supplement 7—source data 1. [file elife-99572-fig4-figsupp7-data1.zip › Figure 4ΓÇöfigure supplement 7F_right_Labelled.tiff]

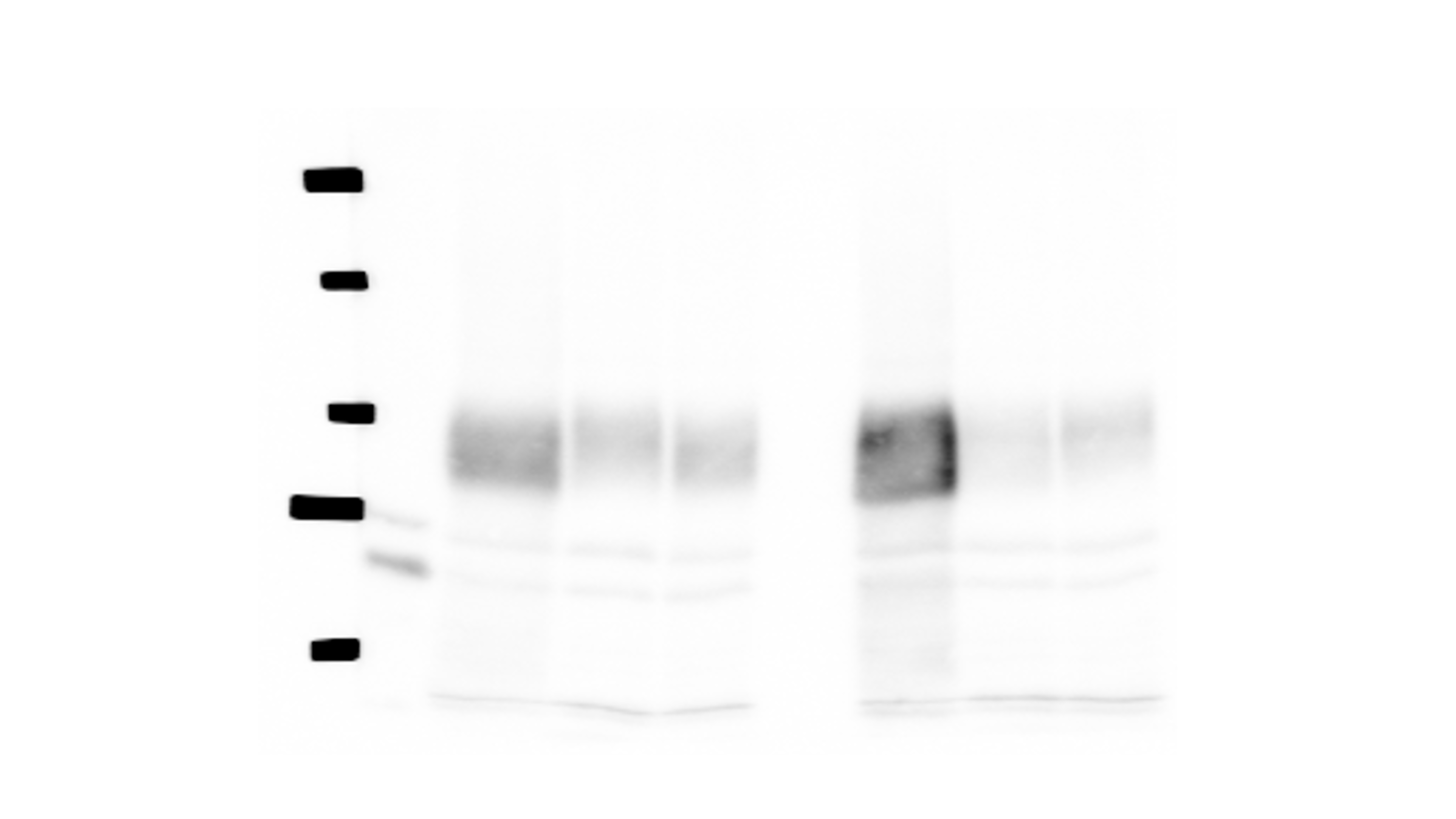

Supplement: Figure 4—figure supplement 7—source data 1. [file elife-99572-fig4-figsupp7-data1.zip › Figure 4ΓÇöfigure supplement 7F_right_Original.tiff]

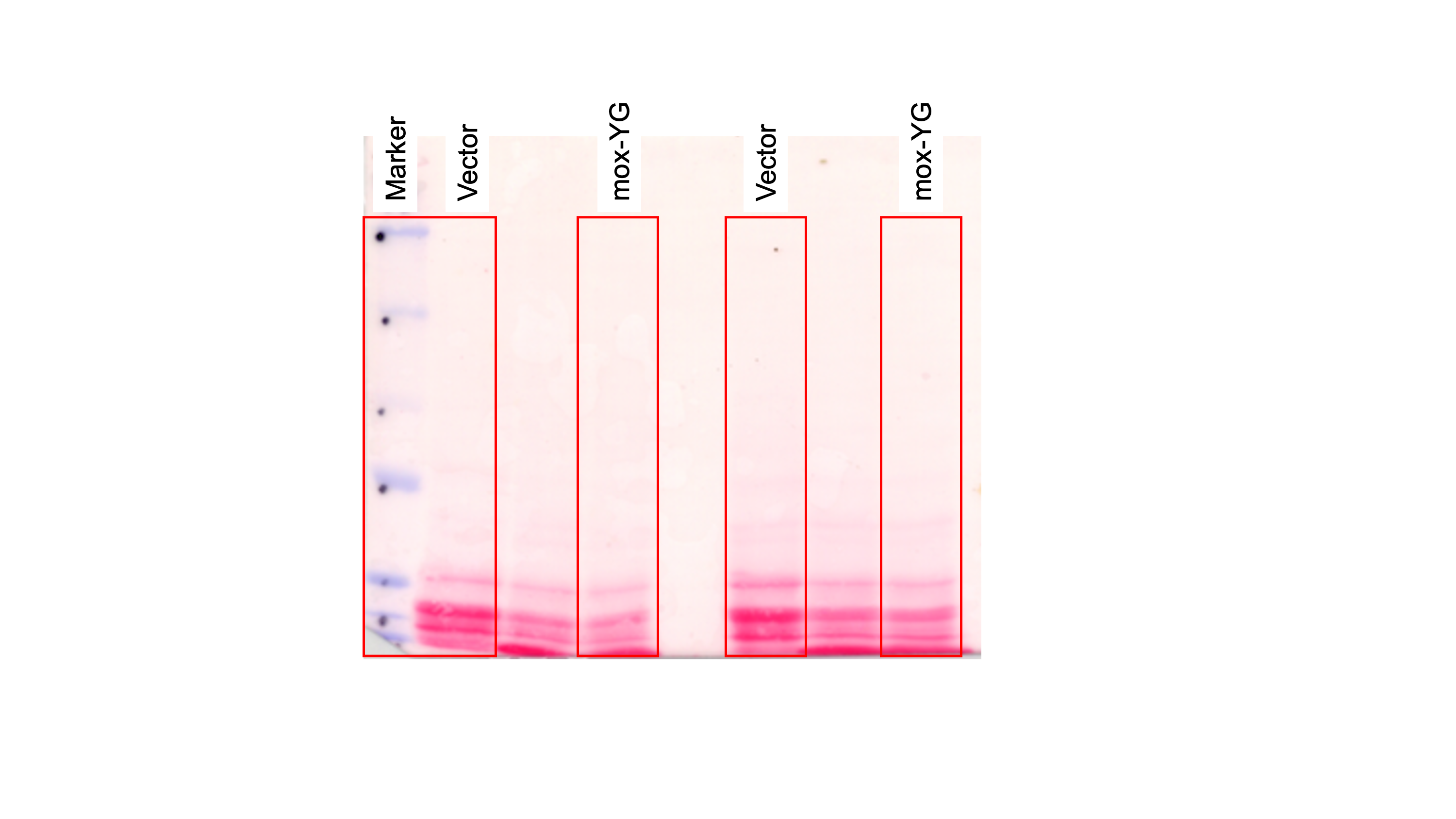

Supplement: Figure 4—figure supplement 7—source data 1. [file elife-99572-fig4-figsupp7-data1.zip › Figure 4ΓÇöfigure supplement 7F_left_Labelled.tiff]

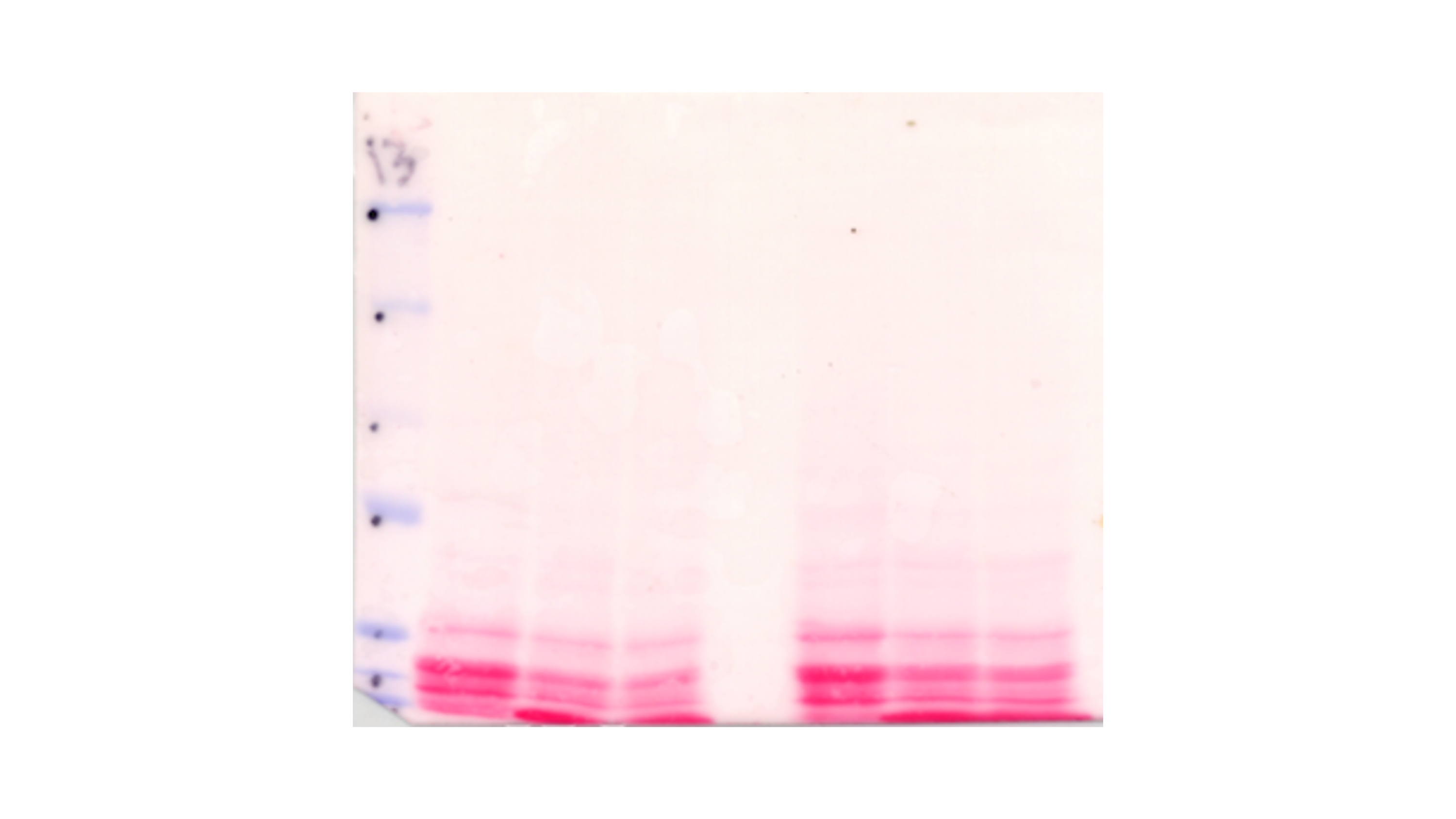

Supplement: Figure 4—figure supplement 7—source data 1. [file elife-99572-fig4-figsupp7-data1.zip › Figure 4ΓÇöfigure supplement 7F_left_Original.tiff]

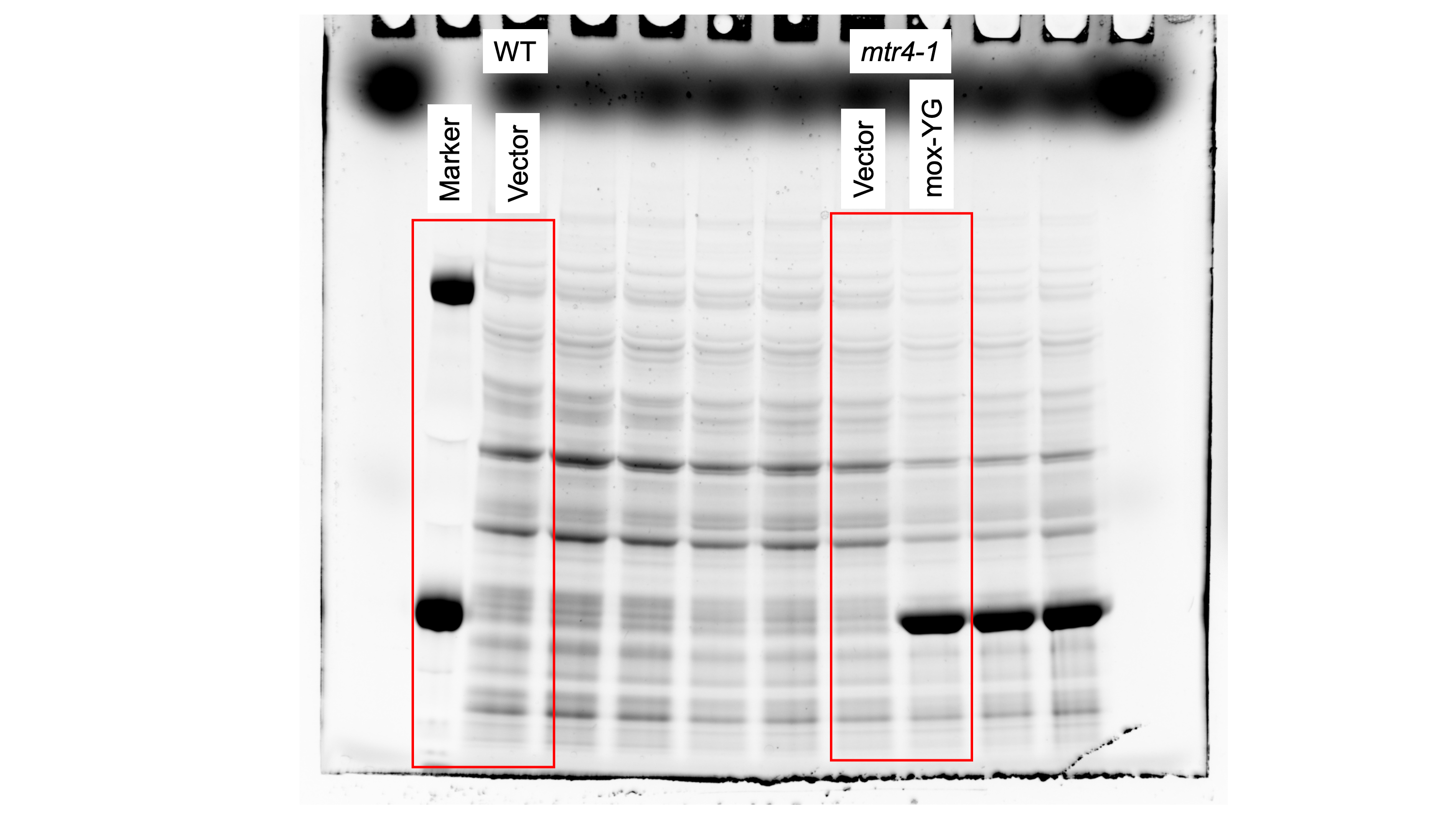

Supplement: Figure 5—figure supplement 5—source data 1. [file elife-99572-fig5-figsupp5-data1.zip › Figure 5ΓÇösource data 1/Figure 5ΓÇöfigure supplement 5C_Labelled.tiff]

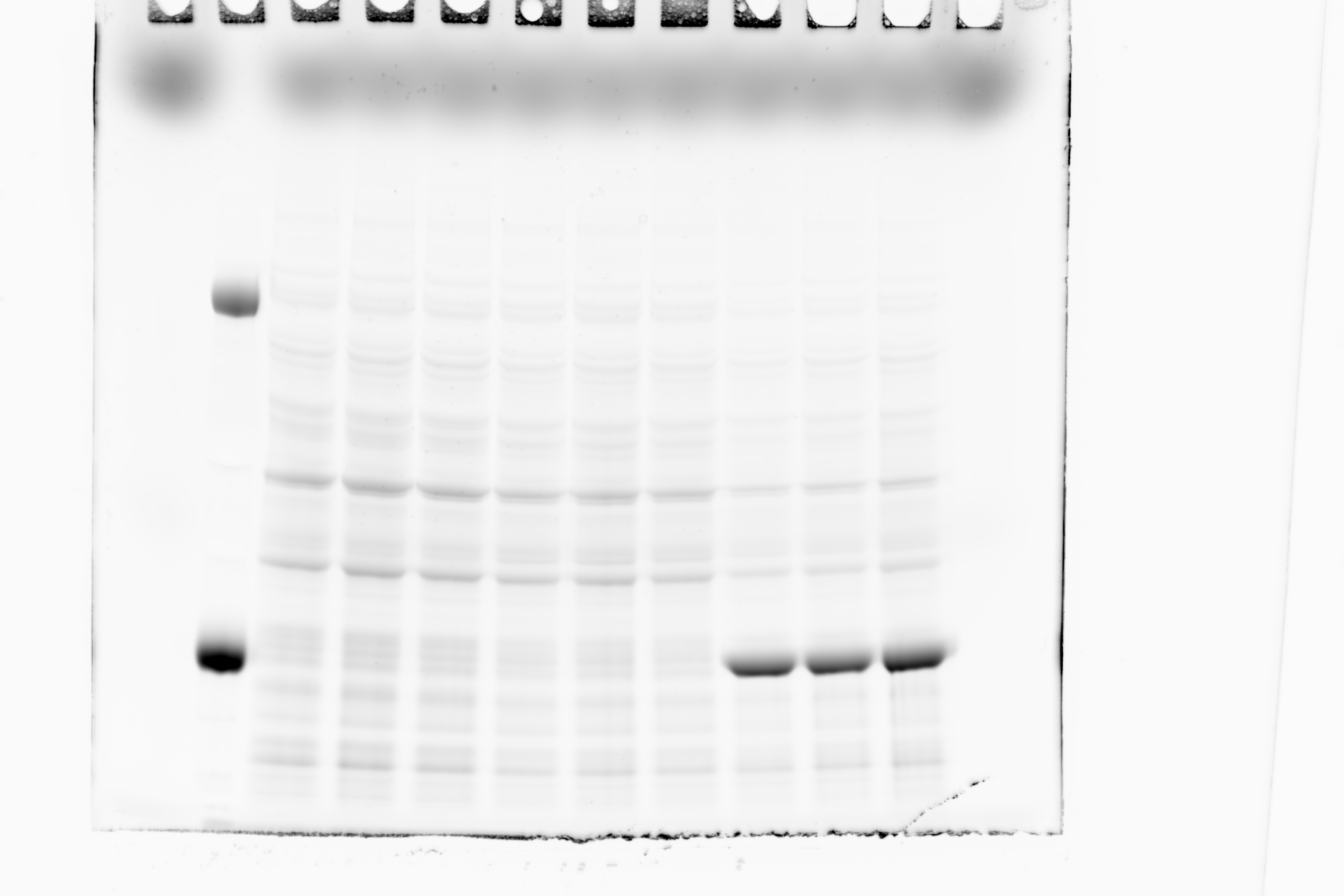

Supplement: Figure 5—figure supplement 5—source data 1. [file elife-99572-fig5-figsupp5-data1.zip › Figure 5ΓÇösource data 1/Figure 5ΓÇöfigure supplement 5C_Original.gel]

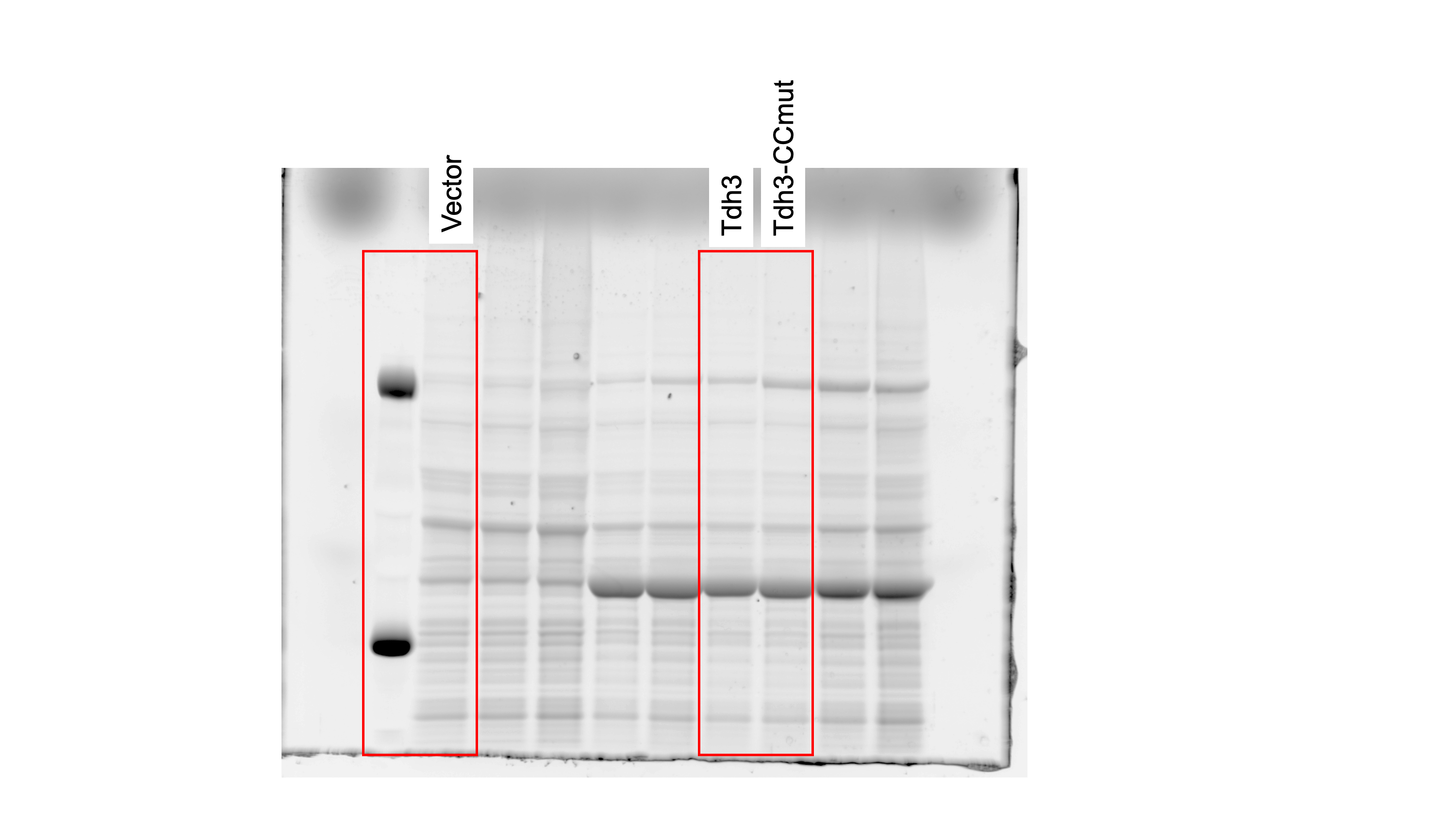

Supplement: Figure 6—figure supplement 1—source data 1. [file elife-99572-fig6-figsupp1-data1.zip › Figure 6ΓÇösource data 1/Figure 6 ΓÇöfigure supplement 1D_1_Labelled.tiff]

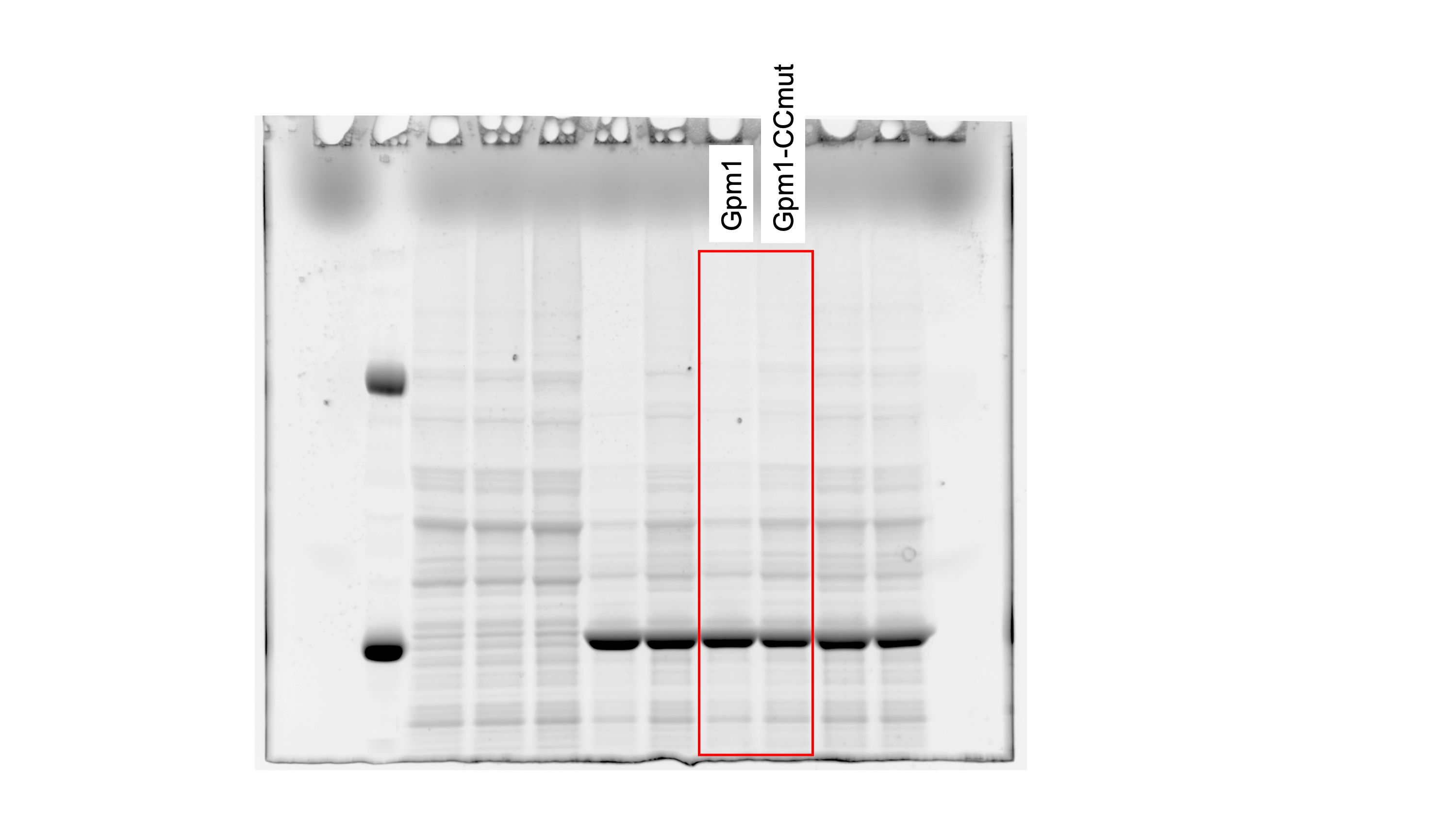

Supplement: Figure 6—figure supplement 1—source data 1. [file elife-99572-fig6-figsupp1-data1.zip › Figure 6ΓÇösource data 1/Figure 6 ΓÇöfigure supplement 1D_2_Labelled.tiff]

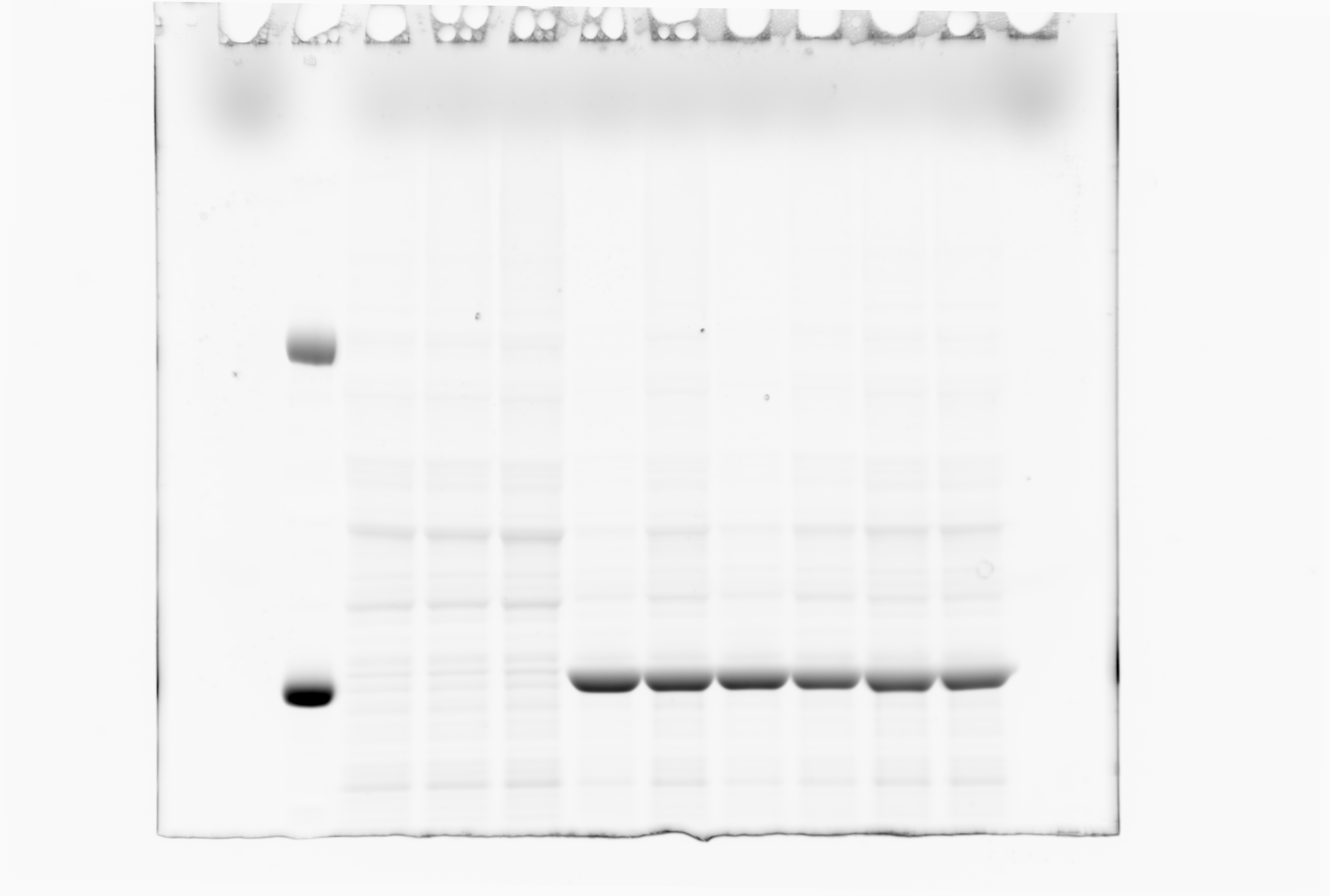

Supplement: Figure 6—figure supplement 1—source data 1. [file elife-99572-fig6-figsupp1-data1.zip › Figure 6ΓÇösource data 1/Figure 6 ΓÇöfigure supplement 1D_1_Original.tiff]

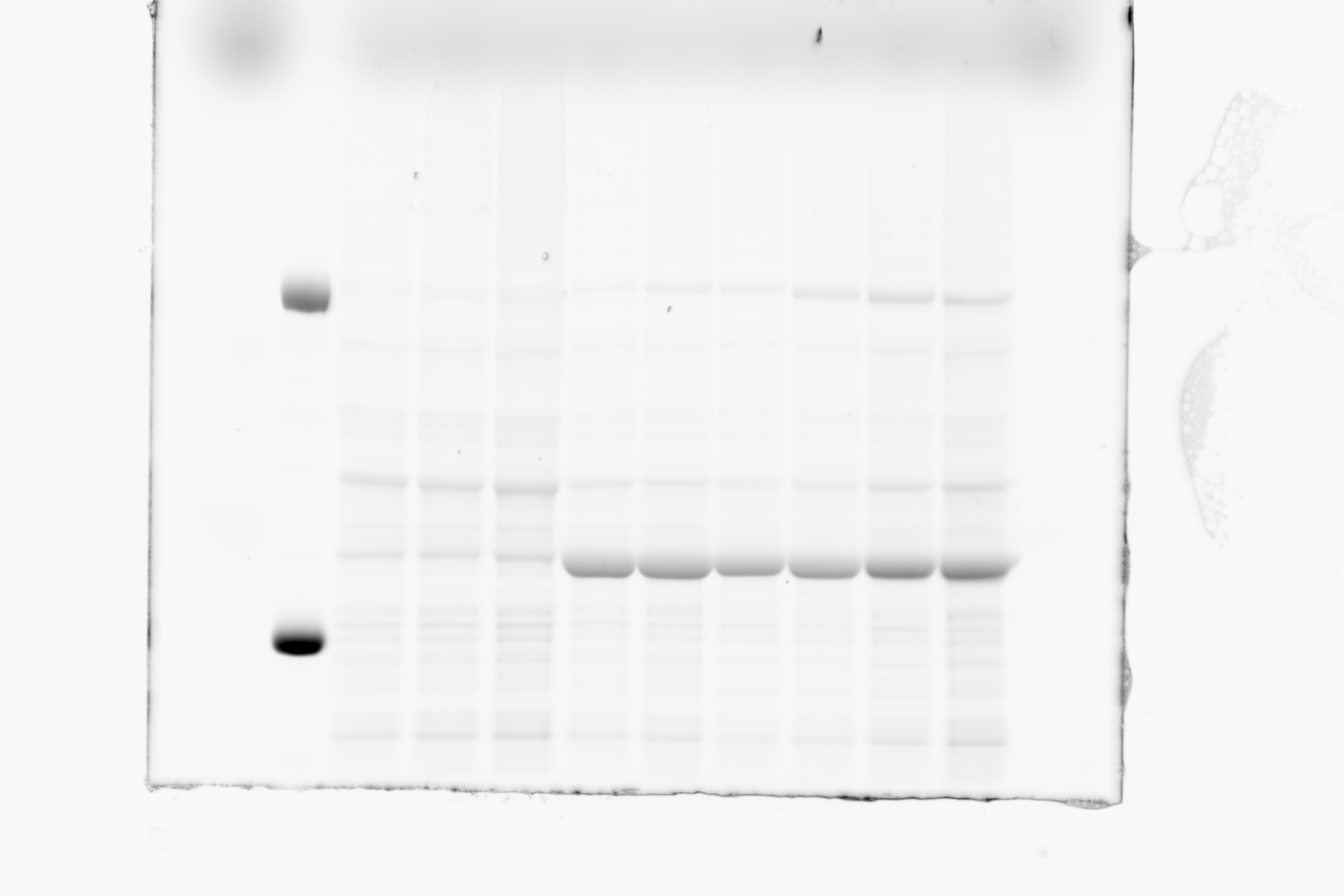

Supplement: Figure 6—figure supplement 1—source data 1. [file elife-99572-fig6-figsupp1-data1.zip › Figure 6ΓÇösource data 1/Figure 6 ΓÇöfigure supplement 1D_2_Original.tiff]
